# Dynamic stability of sequential stimulus representations in adapting neuronal networks

**DOI:** 10.3389/fncom.2014.00124

**Published:** 2014-10-22

**Authors:** Renato C. F. Duarte, Abigail Morrison

**Affiliations:** ^1^Institute of Neuroscience and Medicine (INM-6) and Institute for Advanced Simulation (IAS-6), Jülich Research Center and JARAJülich, Germany; ^2^Bernstein Center Freiburg, Albert-Ludwig University of FreiburgFreiburg im Breisgau, Germany; ^3^Faculty of Biology, Albert-Ludwig University of FreiburgFreiburg im Breisgau, Germany; ^4^School of Informatics, Institute of Adaptive and Neural Computation, University of EdinburghEdinburgh, UK; ^5^Faculty of Psychology, Institute of Cognitive Neuroscience, Ruhr-University BochumBochum, Germany

**Keywords:** stimulus representation, synaptic plasticity, excitation/inhibition balance, transient dynamics, asynchronous irregular states, online computation

## Abstract

The ability to acquire and maintain appropriate representations of time-varying, sequential stimulus events is a fundamental feature of neocortical circuits and a necessary first step toward more specialized information processing. The dynamical properties of such representations depend on the current state of the circuit, which is determined primarily by the ongoing, internally generated activity, setting the ground state from which input-specific transformations emerge. Here, we begin by demonstrating that timing-dependent synaptic plasticity mechanisms have an important role to play in the active maintenance of an ongoing dynamics characterized by asynchronous and irregular firing, closely resembling cortical activity *in vivo*. Incoming stimuli, acting as perturbations of the local balance of excitation and inhibition, require fast adaptive responses to prevent the development of unstable activity regimes, such as those characterized by a high degree of population-wide synchrony. We establish a link between such pathological network activity, which is circumvented by the action of plasticity, and a reduced computational capacity. Additionally, we demonstrate that the action of plasticity shapes and stabilizes the transient network states exhibited in the presence of sequentially presented stimulus events, allowing the development of adequate and discernible stimulus representations. The main feature responsible for the increased discriminability of stimulus-driven population responses in plastic networks is shown to be the decorrelating action of inhibitory plasticity and the consequent maintenance of the asynchronous irregular dynamic regime both for ongoing activity and stimulus-driven responses, whereas excitatory plasticity is shown to play only a marginal role.

## 1. Introduction

As we navigate the world, we are continuously exposed to dynamic and highly complex streams of multimodal sensory information, which we tend to perceive as a series of discrete and coherently bounded sub-sequences (Schapiro et al., 2013). While these *perceptual events* (Zacks and Tversky, 2001; Zacks et al., 2007) are unfolding, active representations are maintained and ought to be sufficiently discernible by the activity of the processing networks, its attributes being encoded by the distributed responses of specifically tuned neuronal populations that are transiently associated into coherent ensembles (von der Malsburg et al., 2010; Singer, 2013).

Neocortical circuits must therefore self-organize to dynamically adopt relevant representations in a stimulus- and state-dependent manner, while maintaining the necessary sensitivity to allow global shifts in representational space when sudden event transitions occur. The primary source of such sensitivity in stereotypically sparse recurrent networks, such as those encountered in the neocortex, lies in the balance of excitation and inhibition (Tsodyks and Sejnowski, 1995; van Vreeswijk and Sompolinsky, 1998; Haider et al., 2006). This endows the network with a stable, ongoing background activity, characterized by low-frequency, asynchronous and irregular firing patterns, under stationary conditions (Gerstein and Mandelbrot, 1964; Softky and Koch, 1993; Destexhe et al., 2003; Stiefel et al., 2013). Such a dynamic state provides the substrate for complex responses to external stimuli to develop as a transient spatiotemporal succession of network states (Mazor and Laurent, 2005; Rabinovich et al., 2008; Buonomano and Maass, 2009). External stimuli act as perturbations of the stable ongoing activity, causing transient and variable disruptions of local E/I balance, which necessarily influence the resulting network states. Furthermore, most real world stimulus events occur sequentially and not in isolation. Consequently, the quality of the dynamic representations and characteristics of the resulting *neural trajectories* is highly related to the circuit's ability to adaptively remodel and refine its functional connectivity in an experience-dependent manner so as to counteract the targeted disruptions and acquire the relevant structure of the input stimuli.

Although there is a great variety of biophysical mechanisms involving activity-dependent modifications of various components at different spatial and temporal scales, it is widely acknowledged that the synapse is the primary locus of functional adaptation in the cortex (Abbott and Nelson, 2000), with synaptic modifications providing the basis of learning and memory and allowing purposeful computations to take place. While constituting a diverse set, comprising operations over variable dynamic ranges and involving a multitude of possible functional roles, cortical synapses can be broadly categorized based on the nature of source and target neurons they connect and the effect they exert (excitatory or inhibitory). Understanding and exploring the possible adaptation mechanisms involved in each of these sub-classes and how they interact is important to understand the nature of neural computation. It is reasonable to assume that the required flexibility to support highly complex cognitive computations, relies on the effects of the combined action of multiple, synergistic, plasticity mechanisms.

There is a large body of experimental evidence and theoretical investigations concerning adaptation at excitatory synapses. It has long been experimentally observed that, in cortical pyramidal neurons, the magnitude and direction of change in the strength of a synapse is dependent on the relative timing of pre- and post-synaptic spikes, when they occur within a critical coincidence time window (Gustafsson et al., 1987; Markram et al., 1997; Bi and Poo, 1998). The functional implications of this observation for cortical processing and unsupervised, experience-dependent adaptation have since been the subject of intense investigation and have proven capable to account for several important computational features of cortical processing (for reviews, see e.g., Dan and Poo, 2004, 2006; Sjöström and Gerstner, 2010; Markram et al., 2012). However, despite this progress, attempts to endow recurrent networks with the ability to learn the underlying structure of their inputs using excitatory spike timing dependent plasticity have been largely unsuccessful (Kunkel et al., 2011).

In contrast, research on inhibitory synaptic plasticity is still sparse and its computational role somewhat speculative. Given the ubiquity of inhibition in the cortex (~20% of all cortical neurons are inhibitory, see Braitenberg and Schüz, 1998) and its undeniable role in shaping and stabilizing network dynamics and neuronal excitability, the possible functional implications of dynamic inhibition are of great interest, particularly when interacting with other forms of plasticity (see Kullmann et al., 2012; Vogels et al., 2013 for an overview). Progress in this endeavor is hindered by the complexity and diversity of inhibitory neurons, making it technically challenging to obtain reproducible experimental results and difficult to reconcile the available data. Nevertheless, recent evidence shows that, in cortical networks, GABAergic synapses targeting excitatory neurons are also sensitive to temporally coincident pre- and post-synaptic spiking (Holmgren and Zilberter, 2001; Woodin et al., 2003). To capture this phenomenon, Vogels et al. (2011) studied the computational effects of a simplified, symmetric inhibitory STDP rule in the establishment and robust maintenance of detailed balance between excitation and inhibition, both in a feedforward and in a recurrent configuration, showing that it allows the emergence of stimulus selectivity and memory. Apart from these self-organized computational roles of inhibitory plasticity, the mechanism implemented by Vogels et al. (2011) has the interesting property of serving as a homeostatic mechanism. It maintains the post-synaptic firing rate under control by dynamically stabilizing the amount of inhibitory and excitatory drive that the excitatory neurons receive, which is particularly relevant in a situation where the excitatory drive is also dynamic, given the possible interdependence between excitatory and inhibitory synaptic plasticity (Wang and Maffei, 2014).

In this work, we consider the combined influence of timing-dependent synaptic plasticity rules operating on different synapse types and analyse its impact on the stability and diversity of global network dynamics as well as their computational implications for online processing of time-varying input streams. Following the general framework of reservoir computing (Lukoševicius and Jaeger, 2009), we explore the properties of information processing based on robust transient dynamics and analyse the influence of plasticity on the development and maintenance of dynamic stimulus representations, while maintaining a stable global dynamics. For that purpose, we implement numerical simulations of biologically realistic networks of leaky integrate-and-fire neurons which incorporate excitatory and inhibitory plasticity, combining well characterized phenomenological models of synaptic plasticity that take into account relevant physiological observations (Morrison et al., 2008; Vogels et al., 2011).

We begin by demonstrating that the balancing effects of these synaptic plasticity rules actively maintain an asynchronous irregular pattern of ongoing, background activity throughout the network, over a much broader range of parameters, compared with networks whose synapses are fixed and static. Furthermore, we establish a relation between dynamical states characterized by a regular, synchronous population firing pattern (which are mostly abolished by the action of plasticity) and a decreased capacity to process generic time-varying input streams, reinforcing the claim that the modulatory actions of plasticity have an important impact on computational performance.

Subsequently, we assess the features of population responses to sequentially occurring stimulus events, modeled as sudden spike bursts across variable numbers of afferent neurons providing a “wake-up” call (Sherman, 2001a) to stimulus-specific sub-populations via targeted, brief disruptions of balance. This stimulus is chosen primarily to be simple but disruptive rather than to model a particular cortical input, however in the following we refer to this type of stimulus as thalamic due to a similarity to the thalamic burst mode of firing (Ramcharan et al., 2000; Sherman, 2001b; Bruno and Sakmann, 2006). We show that the properties of ongoing, dynamic stimulus representations are naturally bound to the stimulus features and the strength of the disruption, but also to the characteristics of the ongoing network activity, that sets the dimensionality of the embedding space over which dynamic representations can unfold. By improving the stability of this ongoing activity and the robustness and reproducibility of response transients, plasticity is shown to benefit the quality of the representations, necessary for subsequent processing by downstream cortical regions.

In the final section of the results, we attempt to disentangle the roles played by the two analyzed plasticity rules in the development of adequate stimulus representations, concluding that the quality of such representations is largely dependent on the decorrelating actions of inhibitory STDP, which results in the maintenance of AI-type activity across the network. The role played by excitatory STDP only provides a marginal advantage compared to static networks, which is an unexpected result leading us to draw some tentative conclusions and opening up a new set of questions to be addressed in future studies.

## 2. Materials and methods

In this section, we describe the equations used to model neuronal and synaptic dynamics, the characteristics of the input-dependent tasks, as well as the methods used for numerical simulations and data analysis. A summarized, tabular description of all the models and model parameters used throughout this study is available in the Supplementary Materials.

### 2.1. Network

#### 2.1.1. Neuron and synapse models

The networks we analyse are composed of *N* = 10000 leaky integrate-and-fire neurons (of which *N*^E^ = 0.8 *N* are excitatory and *N*^I^ = 0.2 *N* inhibitory) with fixed voltage threshold (Tuckwell, 1988) and conductance-based synapses (Koch, 2004), which capture a broad range of intrinsic properties shared by cortical neurons.

Synaptic interactions between the neurons are modeled as transient conductance fluctuations, so the sub-threshold membrane potential *V_i_* of the *i*-th neuron (*i* = 1, …, *N*_α_) belonging to population α is given by:

(1) $$C_{\text{m}}\frac{dV_{i}(t)}{dt}=g_{\text{leak}}\left(V_{\text{rest}}-V_{i}(t)\right)+I_{i}^{\alpha \text{E}}(t)+I_{i}^{\alpha \text{I}}(t)+I_{i}^{\alpha \text{X}}(t)$$

where *I*^α*Y*^_*i*_ is the sum of all synaptic currents generated by the pre-synaptic neurons of neuron *i* in population *Y*. The total synaptic input current onto neuron *i* is thus the sum of the individual contributions of excitatory (glutamatergic, AMPA-type) synapses (E), inhibitory (GABAergic) synapses (I), and pre-synaptic sources from outside the network (X). The latter models cortical background activity and is assumed, for simplicity, to be non-selective and stochastic, with fixed rate ν_X_. When applicable, some neurons belonging to discrete sub-populations receive additional, patterned external stimulation (see Section 2.4). The synaptic current induced in a post-synaptic neuron *i* in population α when a pre-synaptic neuron *j* in population β fires is given by:

(2) $$I_{ij}(t)=g_{ij}(t)(V_{\beta }-V_{i}(t))$$

where *V*_β_ is the equilibrium/reversal potential of the corresponding synapse. The time course of the synaptic conductance *g_ij_*(*t*) is modeled as an instantaneous rise triggered by each pre-synaptic spike, followed by an exponential decay:

(3) $$\frac{dg_{ij}(t)}{dt}=-\frac{g_{ij}(t)}{\tau_{\beta }}+{\bar{g}}^{\beta }w_{ij}(t)\sum_{t_{j}}\delta \left(t-t_{j}-d\right)$$

where δ(.) is the Dirac delta function, *t_j_* are the spike times of the pre-synaptic neuron and *d* refers to the conduction delay, which is set to be constant and equal for all synapses, with the value of 1.5 ms.

The peak amplitude of the conductance transient, which determines the “strength” of the synapse, is the product of a constant scaling factor *g*^β^, whose value depends on the synapse type and is used to set the scale of the synaptic conductance, and a dimensionless variable *w_ij_*, assumed to be dynamic in synapses subjected to activity-dependent adaptation (see Section 2.2) and whose initial value is drawn from a Gaussian distribution with mean μ^β^ and standard deviation σ^β^, which we set to 1 and 0.25, respectively, leading to narrowly distributed initial peak conductances centered around *g* for every synapse type. All synaptic events originating from outside the network are assumed to be excitatory, with the same reversal potential, peak conductance and time constant as recurrent excitatory synapses, i.e., *V*_X_ = V_E_, *g*^X^ = *g*^E^ and τ_X_ = τ_E_.

Following Kumar et al. (2008b), we quantify the effective balance between excitation and inhibition as the approximate ratio of total charges induced at rest:

(4) $$g=\frac{\langle g^{\alpha \text{I}}\rangle \tau_{\text{I}}|V_{\text{rest}}-V_{\text{I}}|}{\langle g^{\alpha \text{E}}\rangle \tau_{\text{E}}|V_{\text{rest}}-V_{\text{E}}|}$$

with 〈*g*^αI^〉 = μ^I^*g*^I^ and 〈*g*^αE^〉 = μ^E^*g*^E^. Under these conditions, and with all other synaptic parameters fixed and set as described below in Section 2.3.1, we determine the initial value of *g* to be 0.29γ where γ is the ratio of absolute peak conductances.

The parameter values of the neurons are homogeneous across neuron types and are kept fixed throughout. They were chosen for their biological plausibility and consistency with the experimental literature and previous modeling work (e.g., Compte et al., 2000; Meffin et al., 2004; Kumar et al., 2008b; Vogels et al., 2011; Yger et al., 2011). A complete description of the parameters and their values can be found in the Supplementary Materials.

#### 2.1.2. Network architecture

All the network neurons are laid on the integer points of a 2-dimensional 100 × 100 regular grid lattice, with periodic boundary conditions and are sparsely and randomly connected. The probability of connection between a target neuron in population α and a source neuron in population β is set to 0.1 for αβ ∈ {EE, EI, IE, II}, such that, on average, each neuron in the network receives a total of *K*^E^ = 0.1 · *N*^E^ excitatory and *K*^I^ = 0.1 · *N*^I^ inhibitory, randomly chosen, synaptic inputs from the local network, along with *K*^X^ synaptic inputs from outside the network. It is generally assumed that the number of background synapses from external cortical sources, comprising patchy long-range input from the same cortical area as well as input from distant cortical areas (Braitenberg and Schüz, 1998; Kumar et al., 2008a; Kremkow et al., 2010) lies in the same range as the number of local, recurrent excitatory connections, so we set *K*^X^ = *K*^E^ (Brunel, 2000).

This network structure is relevant mostly for the purpose of visualization when patterned stimuli are delivered to specific, spatially clustered neuronal populations, given that no additional spatial constraints are imposed on the connectivity structure. Furthermore, in networks shaped by plasticity, the connectivity structure remains unaltered, i.e., plasticity modifies the strength of existing connections only and does not create new synapses or destroy existing ones.

### 2.2. Synaptic plasticity

In the following, we assume that synapses targeting inhibitory neurons (II and IE) are static, whereas synapses targeting excitatory neurons (EI and EE) are sensitive to pre- and post-synaptic spike times. Although there is increasing evidence for the existence of timing-dependent adaptation mechanisms in synapses within inhibitory populations (Lamsa et al., 2010) and from excitatory to inhibitory neurons (Lu et al., 2007) (II, IE, respectively), their precise mechanisms are highly dependent on the target neuron type, which constitutes an added source of heterogeneity and complexity. Furthermore, in most of our analysis, we assume that the most relevant activity is that which can be propagated to downstream regions, conveying the relevant information for additional processing. For that reason, we focus our attention on the dynamics of the excitatory population, given the known locality of inhibitory connections.

In all experiments on networks incorporating plasticity, synapses are continually plastic. For convenience, notation brevity and consistency, plasticity modifications are not applied directly to the synaptic “strength” (i.e., to the peak amplitude of the conductance transient), but instead, to the dimensionless variable *w_ij_* which is subsequently rescaled by a constant factor (see Equation 3).

Both types of plasticity used can be expressed in terms of a synaptic trace variable defined for each neuron that is incremented by 1 at each spike and decreases exponentially in between spikes:

(5) $$\frac{dx_{i}(t)}{dt}=-\frac{x_{i}}{\tau_{\text{p}}}+\sum_{t_{i}}\delta (t-t_{i})$$

#### 2.2.1. Excitatory STDP

We term the learning rule applied to the recurrent synapses among the excitatory population excitatory spike-timing dependent plasticity (eSTDP) and adopt the formalism proposed in van Rossum et al. (2000):

(6) $$\Delta w^{\text{EE}}=\{\begin{array}{ll} \lambda \exp(-|\Delta t|/\tau_{\text{p}}), & \text{if}\Delta t>0\\ \alpha_{\text{ep}}\lambda w^{\text{EE}}\exp(-|\Delta t|/\tau_{\text{p}}), & \text{if }\Delta t\leq 0 \end{array}$$

where |Δ*t*| = *t^f^_i_* − *t^f^_j_* is the absolute difference between a specific pair of spikes of pre-synaptic neuron *j* and post-synaptic neuron *i* and τ_p_ is the time window for potentiation and depression, which we set to be equal (τ_p_ = 20 ms, following experimental data obtained by Bi and Poo, 1998). The parameter λ sets the magnitude of individual modifications (i.e., the learning rate) and α_ep_ determines the asymmetry between the amount of potentiating and depressing changes. The update rule can be re-written in a differential form that depends on the synaptic trace variables:

(7) $$\frac{dw_{ij}}{dt}=\alpha_{\text{ep}}\lambda w^{\text{EE}}x_{i}(t)\delta (t-t_{j}^{f})+\lambda x_{j}(t)\delta (t-t_{i}^{f})$$

with all propagation delays considered to be dendritic, i.e., spike times are taken at the synapse and no autapses are allowed. The pre- and post-synaptic spikes are paired in an all-to-all scheme (see Morrison et al., 2008).

This “hybrid” learning rule is additive for potentiation and multiplicative for depression, thus incorporating some of the most relevant experimental observations (Bi and Poo, 1998). Importantly, it gives rise to unimodal weight distributions similar to those observed experimentally in the presence of uncorrelated input, while retaining the ability to develop multimodal distributions depending on the input correlation structure.

#### 2.2.2. Inhibitory STDP

We apply the inhibitory spike-timing dependent plasticity (iSTDP) proposed in Vogels et al. (2011) to the weights of synapses between inhibitory and excitatory neurons. The general premise is that pre- and post-synaptic firing that occurs within the relevant coincidence time window (τ_p_) should always lead to synaptic potentiation, regardless of the temporal ordering of the spike pair, whereas isolated pre-synaptic spikes lead to synaptic depression.

This rule can be given in terms of the synaptic trace variables (Equation 5) as:

(8) $$\frac{dw_{ij}}{dt}=\eta \left(x_{i}(t)-\alpha_{\text{ip}}\right)\delta \left(t-t_{j}^{f}\right)+\eta x_{j}(t)\delta \left(t-t_{i}^{f}\right)$$

where η is a constant learning rate. The parameter α_ip_ sets the amount of synaptic depression upon a pre-synaptic spike and has the value α_ip_ = 2ρ_0_τ_p_, where ρ_0_ is a constant which serves the homeostatic purpose of stabilizing the post-synaptic neuron's firing rate (for further details, see Vogels et al., 2011).

### 2.3. Constraining model parameters

#### 2.3.1. Initial synaptic strengths

Synaptic strengths are adjusted such that the ongoing, background network activity, prior to any patterned input stimulation, mimics the statistics of cortical background activity: inhibition dominated (i.e., *g*^I^/*g*^E^ > 1), low rate (1–20 spikes/s), irregular single neuron firing (CV_ISI_ ≃ 1) and asynchronous population activity (low average pairwise correlations) (Brunel, 2000; Destexhe et al., 2001; Meffin et al., 2004) (see Section 3.1 and **Figure 2**).

For that purpose, we started by setting the constant background firing to a low rate of ν_X_ = 5 spikes/s and tuned *g*^E^ and *g*^I^ to obtain the desired activity statistics. This resulted in *g*^E^ = 1.8 nS for excitatory synapses, which leads to an EPSP amplitude of around 1.46 mV at rest and *g*^I^ = γ*g*^E^ = 21.6 nS for inhibitory synapses, leading to IPSP amplitudes of around −1.14 mV at rest, where γ = 12 determines the absolute strength of inhibition relative to excitation. This parameter combination results in self-consistent firing rates, whereby each neuron fires with a rate approximately equal to the population rate and to the external rate (ν_*i*_ = ν_net_ = ν_X_). It also provides a reasonable match to experimental data: the ratio of IPSP to EPSP amplitudes at rest is ≃ 0.78 which lies within the range measured in the cortex (Matsumura et al., 1996). Additionally, the mean coefficient of variation of the single neurons' interspike intervals (CV_ISI_) is around 1.1 and their mean membrane potential approximately −62 mV (Destexhe et al., 2003).

#### 2.3.2. Plasticity

We take a similar approach to tune the parameters of the plasticity rules. We wish to obtain similar population dynamics in networks whose synapses are subjected to adaptation and in networks whose synapses are fixed, when driven by uncorrelated background input. To constrain the parameter ranges, we assume the plasticity rules are independent and determine their parameters individually.

In order to allow the effects of the two plasticity mechanisms to balance, we set the rate of synaptic modifications to be the same in each case, i.e., λ = η = 0.01. The only free parameter of iSTDP left to tune is the target firing rate, ρ_0_. As mentioned by Vogels et al. (2011), it is quite convenient to control the firing rate with a single parameter, since we can tune the network to a desired operating point by simply setting the desired rate. For consistency, we set ρ_0_ = 5 spikes/s, to match the average population firing rate displayed by a static network.

To determine the remaining free parameter of the eSTDP rule, α_ep_ (the asymmetry parameter) we look at the equilibrium dynamics of the weight update. We wish to obtain an equilibrium weight distribution whose mean is equal to the mean static weight, i.e., 1.8 nS and an equilibrium firing rate close to the population rate in the static case. Note that, when iSTDP is present, a specific firing rate can be readily achieved as described above. In the absence of iSTDP, the desired features are achieved for a value α_ep_ ≃ 0.92 (data not shown).

### 2.4. Input stimuli

**Figure 1:**
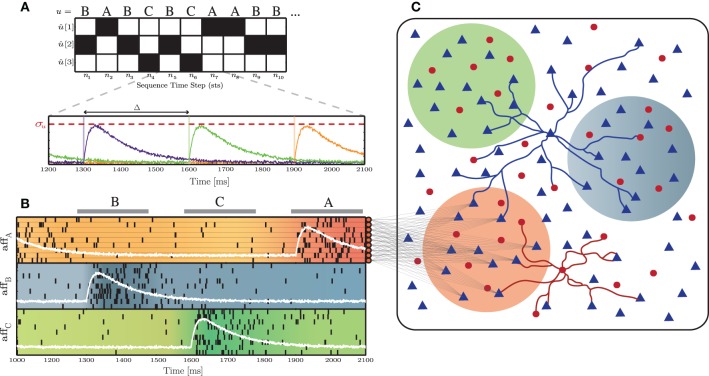
**Schematic representation of the input and network structure used throughout this study**. **(A)** Depiction of the stimulus generation process, converting a symbolic stimulus sequence (*u*), composed of *k* = 3 different, randomly ordered stimuli (*S* = *A, B, C*), into the *k*-dimensional binary representation *û*, over the course of *n* = 10 sequence time steps. These are subsequently transformed into *k* independent signals (*s*_*k*_, each corresponding to a specific color trace in the bottom panel), with a period Δ and a peak amplitude σ_*u*_. **(B)** Each input signal *s*_*k*_(*t*) (white lines) determines the firing rate of *N*_aff_ = 10 afferent neurons. **(C)** Each afferent neuron projects to a subset of γ_*u*_ × *N*^E^ excitatory neurons in the main network. The color of the sub-populations indicates the corresponding input stream.

A sequence of stimuli was delivered to specific sub-populations (Figure 1C), in order to perturb the stable background activity. We call these input stimuli “thalamic,” to differentiate them from the unspecific, background stimulation, referred in Section 2.1.1, however an accurate modeling of thalamic activity is not aimed at in this study. Each stimulus was assigned an arbitrary abstract label and subsequently converted into a set of spike trains, according to the following process: a stimulus sequence (*u* = σ_1_, σ_2_, …, σ_*T*_) of a predefined length (*T*) was built by randomly drawing σ_*i*_ from a set of *k* different stimuli *S*_*j*_ ∈ *S*, with 1 ≤ *j* ≤ *k*. Each successive σ_*i*_ was then converted into a *k*-dimensional binary vector *û*, where *û*_*n*_[*j*] = 1 if *u*_*n*_ = *S_j_*, for *n* = 1, …, *T*. This binary representation was also used as the target output to train the readout units in a classification task (see Section 2.5.3.1). From the resulting input streams (*û*_*n*_), *k* independent signals were generated, according to:

(9) $$s_{k}(t)=\frac{1}{\sigma_{u}}\left({\hat{u}}_{n}[k]\times \delta (t-n\Delta )\right)*g$$

where σ_*u*_ determines the signal's peak amplitude and Δ corresponds to the period of the input sequence, i.e., the duration of each stimulus presentation plus the inter-stimulus interval, assuming regularity of input unit length. The function *g* is a bi-exponential kernel:

(10) $$g(s)=exp(-s/\tau_{r})-exp(-s/\tau_{d})$$

with rise time τ_*r*_ = 50 ms and decay time τ_*d*_ = 150 ms (see Figure 1A for a schematic depiction of this input generation process). These independent signals were used to determine the time-dependent firing rates of inhomogeneous Poisson processes, in order to generate *N*_aff_ input spike trains for each signal *s_k_*. The peak amplitude of the signal thus corresponds to the peak firing rate of a spike burst. Finally, a constant value of 2 spikes/s background activity along with a small amount of Gaussian white noise was added to the signals *s*_*k*_(*t*). The resulting input structure is depicted in Figure 1B.

For the experiments in Section 3.2, we use *k* = 3, randomly drawn, independent stimulus classes. In total, *T* = 3300 stimulus samples (comprising 1100 samples of each stimulus class) are presented to the networks. The first 300 samples and corresponding network responses are considered to represent an initial “entraining” period and so are discarded from the analysis. The duration of each stimulus presentation is fixed and set to 200 ms, followed by a 100 ms-long inter-stimulus interval, resulting in a total analyzed simulation time of 900 s. A similar protocol is used in the experiments in Section 3.3, the difference being that the value of *k* is varied. In such cases we use 1100*k* stimulus samples and discard the first 100*k* samples.

### 2.5. Data analysis

#### 2.5.1. Global network dynamics

In order to properly characterize and compare the dynamic network states in different conditions, we need to adequately quantify the population activity. This analysis focuses on 3 main properties, the average population firing rate, the degree of synchrony and the degree of irregularity of network states.

***2.5.1.1. Irregularity***. The degree of irregularity of population spiking activity is determined by the coefficient of variation of the interspike intervals (ISI) of each neuron's spike trains, averaged across all neurons in the population:

(11) $${\text{CV}}_{\text{ISI}}=\langle \frac{\sigma_{i}^{\text{ISI}}}{\mu_{i}^{\text{ISI}}}\rangle$$

where 〈.〉 denotes the average over all neurons, μ^ISI^_*i*_ and σ^ISI^_*i*_ denote the mean and standard deviation of the ISIs of neuron *i*. The CV_ISI_ provides a good measure of spike train variability over time scales on the order of the mean ISI. An irregularly spiking neuronal population will have CV_ISI_ close to 1 (a value of exactly 1 corresponds to Poissonian firing). CV_ISI_ values close to 0 indicate a regular spiking pattern, whereas values much larger than 1 indicate a bursting firing profile.

***2.5.1.2. Synchrony***. The degree of synchrony is quantified by the average pairwise correlation coefficient over 500 randomly sampled, disjoint, neuronal pairs (see, e.g., Kumar et al., 2008b):

(12) $${\text{CC}}_{ij}=\langle \frac{\text{Cov}(C_{i},C_{j})}{\text{Var}(C_{i})\text{Var}(C_{j})}\rangle$$

where 〈.〉 denotes the average over all pairs, *C*_*i*_ and *C*_*j*_ represent the spike counts of neurons *i* and *j*, computed by counting the spikes occurring within successive time bins of width 1 ms.

***2.5.1.3. AI score***. To allow a simpler visualization of the regions encompassing AI-type activity (see Section 3.1), we introduce an additional metric that summarizes the main statistical descriptors and provides a graded measure of the *AIness* of the spiking activity, depicted in **Figures 3E,J**. This metric relies on somewhat arbitrary criteria, based on general assumptions, which we establish as the percentage of neurons in the population that fire with a rate ≤20 spikes/s and whose CV_ISI_ ∈ [0.8, 1.5], in conditions where the average pairwise CC ≤ 0.05. Objectively, all the parameter combinations that lie within this range reflect AI-type activity, but may correspond to different sub-types of this regime (Ostojic, 2014), with different computational properties. The highest AI scores, in our analysis, reflect the “classical” states, of homogeneous average firing rates.

#### 2.5.2. Global computational power

We adopt the methods introduced in Maass et al. (2005), Legenstein and Maass (2007) to evaluate the generic computational power of neuronal microcircuits, regardless of the precise nature of the circuit. The general premise underlying this approach is that sufficiently different input streams should cause different internal states and hence lead to different, linearly separable outputs.

For this purpose, we can use a much simpler stimulus than previously described in Section 2.4. Consider the microcircuit *C*, generated with a specific set of parameters, and stimulated by one of a set of *T* = 500 different input streams (fixed spike patterns), each composed of 4 independent Poisson spike trains, at a rate of 20 spikes/s and a duration of 200 ms. The temporal evolution of the system in response to this input pattern is analyzed and stored in a response matrix, obtained by convolving each neuron's spike train with an exponential kernel, with 30 ms time constant and temporal resolution equal to the simulation time step, i.e., 0.1 ms (Section 2.6), in order to capture the effect of each spike on the membrane properties of a readout neuron receiving it. This response matrix is then sampled at time point *t*_0_ = 200 ms, resulting in the *N*-dimensional vector *x*_*u*_(*t*_0_) which contains all the neurons' responses to input pattern *u*, or the circuit state (Maass et al., 2002). The procedure is then repeated for all the spike templates, leading to the formation of the state matrix *X* ∈ ℝ^*N* × *T*^. The rank *r* of the matrix *X*, calculated by singular value decomposition, corresponds to the number of linearly independent columns of *X*, i.e., the number of inputs that are mapped into linearly independent circuit states, thus providing a quantitative descriptor of computational performance, or *kernel quality*. If *r* ≤ *k*, a linear readout should be able to separate *r* classes of inputs (Maass et al., 2005; Legenstein and Maass, 2007).

#### 2.5.3. Stimulus representation

To assess the quality of the input-state mappings, in relation to the underlying dynamical states that the networks achieve under different conditions, we use the following metrics:

***2.5.3.1. Readout classification***. The network responses to a stimulus sequence of length *T* are assembled in a state matrix *X* ∈ ℝ^*N* × *T*^, as described in Section 2.5.2, where each column represents the network state in response to one stimulus. These *T* stimulus-response pairs are subsequently split into train and test samples, with *T*_tr_ = 0.8*T* and *T*_te_ = 0.2*T*. A set of *k* linear readout units are then trained to classify which pattern was presented to the network, using the binary signal *û* as the supervisory signal. We wish to map each *N*-dimensional state pattern (*X*(*t*)) to the corresponding input that triggered it (*û*_*t*_), by minimizing the quadratic error *E*(*Û*, *W*^out^*X*). The synaptic weights from the main network to the readout units (*W*^out^) are obtained by ridge regression, i.e., by solving:

(13) $$W^{\text{out}}=\hat{U}X^{T}{\left(XX^{T}+\alpha^{2}𝕀\right)}^{-1}$$

where *Û* is a *k* × *T*_tr_ matrix combining all the binary target patterns, *X* is the *N* × *T*_tr_ state matrix, 𝕀 is the identity matrix and α is a regularization factor, given by the least squares norm and optimized by 5-fold cross-validation on the training data. In principle, any linear regression method would be applicable, but we chose ridge regularization because of the penalty imposed on the size of the coefficients. It is desirable that the average vector norm of *W*^out^ be kept small so that the output accurately reflects features of the state space, instead of relying on disproportionate amplification of certain dimensions.

The obtained synaptic weights *W*^out^ are then used to classify the state responses to the test sequence. Average classification performance is obtained by applying winner-takes-all on the readout output *y*(*t*) to determine the label assignments and subsequently quantifying the fraction of correctly classified patterns. To obtain a more fine-grained measurement, we also quantify the performance in classifying each of the individual stimulus patterns using the raw readout output and correlating it with the target binary values, using point-biserial correlation coefficient, which is a suitable statistic to estimate the relationship between a dichotomous variable (target values) and a continuous variable (readout output).

***2.5.3.2. Dimensionality reduction and visualization***. Throughout this study, we apply various different methods of dimensionality reduction, which we briefly outline below. It is worth noting that, while for most depictions we chose one particular method, the only criteria that justified this choice was adequate visualization. In every case, several different methods were applied and these results were only included and further discussed if they were consistent across different methods.

These methods are applied to visualize the underlying spatial arrangement of the network states in response to each stimulus pattern (finding structure in the state matrix) and the unfolding trajectory of network states within the time course of single responses to a stimulus, both of which assuming the desirable condition that the input-driven stimulus responses lie within distinct sub-spaces in the *N*-dimensional state space. In the first case, the low-pass filtered population responses to each stimulus are sampled at time *t*_0_ after stimulus onset (typically *t*_0_ = 200 ms, i.e., at the end of each stimulus presentation) and collected in the state matrix *X* ∈ ℝ^*N* × *T*^, following the description in the previous section (Section 2.5.3.1, Readout Classification). The methods of dimensionality reduction are then applied to *X* (*N* features and *T* samples). We tested several different algorithms for this purpose, namely principal component analysis (PCA), linear discriminant analysis (LDA), spectral embedding and isomap embedding. The first two are based on finding low-dimensional embeddings of the data points, using linear projections of the variables that best explain the original, high-dimensional data, either by identifying the directions in feature space that capture most variance in the data (the top eigenvectors of the data covariance matrix or principal components) or by identifying attributes that account for the most variance between labeled classes (LDA is a supervised method). Spectral and isomap embedding methods, on the other hand, are based on non-linear projections of the data seeking low-dimensional representations that maintain the relative distances (Euclidean distances, in our case) between data points. Spectral embedding (also known as Laplacian eigenmaps) constructs a weighted graph representing the data, using an adjacency matrix based on the pairwise distances between data points. The embedding is subsequently obtained by partial eigenvalue (spectral) decomposition of the graph Laplacian (see e.g., Ng et al., 2001; Belkin and Niyogi, 2003 for a more thorough explanation). Isomap embedding also relies on partial eigenvalue decomposition, but applied to a matrix representing the shortest path lengths between a data point and its nearest neighbors (Tenenbaum et al., 2000).

In the second case, we want to reduce the dimensionality of the data along the time course of single responses to a stimulus. For that purpose, we analyse the full response matrix *R*, using the low-pass filtered population responses, with *R* ∈ ℝ^*N* × *D*^, where *D* corresponds to the duration of the stimulus presentation divided by the response time resolution (0.1 ms). We apply techniques that have been previously used to analyse neural data, with a similar goal in mind, namely principal component analysis (PCA) and locally linear embedding (LLE) (see Churchland et al., 2007 for an overview).

***2.5.3.3. Input/output correlations***. To determine to which degree the activity of each input-specific sub-population (see Sections 2.1.2 and 2.4) becomes specialized to a particular input pattern, we compute firing rate histograms of the output of each population *r*_α_(*t*) over many sequential trials with each of the input patterns and determine the time-averaged firing rate of these responses *r*_α_. These histograms are then correlated with the input signals (*s*_*k*_) to obtain the correlation coefficient of signal *k* with population α:

(14) $$C_{k}^{\alpha }=\frac{\langle (s_{k}(t)-{\bar{s}}_{k})(r_{\alpha }(t)-{\bar{r}}_{\alpha })\rangle }{\sigma_{s_{k}}\sigma_{r_{\alpha }}}$$

This procedure allows us to determine the specialization of each population and the impact that each input signal has on each population.

### 2.6. Numerical simulations

All simulations were performed using the NEST simulating environment (Gewaltig and Diesmann, 2007) with an integration resolution of 0.1 ms. Due to the large memory and computing demands, simulations were carried out on large, parallel computing clusters, using the parallelized kernel of NEST (Morrison et al., 2005). All subsequent calculations and data analysis were performed in Python, using the NumPy and SciPy libraries, as well as the Scikit-learn toolbox (Pedregosa et al., 2011).

## 3. Results

### 3.1. Impact of plasticity on network dynamics

Numerous *in vivo* recordings in awake, behaving animals have revealed the prevalence of highly irregular and seemingly noisy firing patterns in neocortical circuits (Softky and Koch, 1993; Stiefel et al., 2013). Sub-threshold fluctuations of the neurons' membrane potentials lead to irregularly timed, low frequency spiking (on the order of 1–20 spikes/s) (Gerstein and Mandelbrot, 1964; Destexhe et al., 2003), whereas at the population level, activity is characterized by a low degree of synchrony, with small pairwise correlations between spike trains (Abeles, 1991; Vaadia and Aertsen, 1992; Shadlen and Newsome, 1994). Collectively, these characteristic features of neural activity are generally termed “Asynchronous Irregular” (AI) states and are assumed to constitute the “ground state” of ongoing cortical activity.

**Figure 2:**
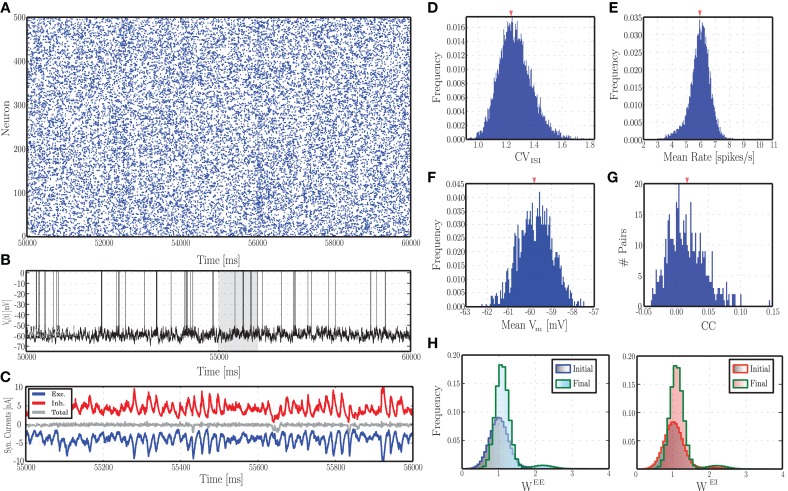
**Characteristics of ongoing activity in a network with excitatory and inhibitory STDP**. **(A)** Raster plot depicting the spiking activity recorded in a subset of 500 randomly chosen excitatory neurons, for a period of 10 s, following an initial equilibration phase of 50 s. **(B)** Example of a randomly chosen single neuron's membrane potential during the same time interval. **(C)** Total excitatory (blue) and inhibitory (red) synaptic currents into the neuron whose membrane dynamics is shown above, during a time period of 2 s (highlighted in gray in **B**). The gray line in the middle corresponds to the total current. **(D–G)** Distributions of the most important descriptors of population activity, namely coefficients of variation of the inter-spike intervals **(D)**, average firing rates **(E)**, mean membrane potentials **(F)**, and pair-wise correlation coefficients (**G**, computed over 500 pairs). These distributions were obtained from the activity of the entire population (not just the neurons depicted in **A**), recorded over a period of 20 s. **(H)** Initial and final synaptic weight distributions for excitatory (left) and inhibitory (right) weights. Note that these distributions refer to the dimensionless variable *w* and not the actual synaptic conductances (see Equation 3).

The mechanisms underlying this activity regime have been the subject of intense investigation and are known to rely mainly on the balance of excitation and inhibition. Analytical studies of random recurrent networks of IF neurons with static current-based synapses have shown that these systems can display a rich set of behaviors, depending on the intensity of external stimulation and the relative strength of excitation and inhibition (van Vreeswijk and Sompolinsky, 1998; Brunel, 2000; Kumar et al., 2008b). In the cortex, this relationship is highly dynamic and the balance required needs to be actively maintained and tuned to allow the network to operate in suitable regimes. Thus, the activity regimes exhibited by networks with plastic synapses is of particular interest. Recently, Vogels et al. (2011) investigated the transition of a network with iSTDP from non-AI to AI regimes in dependence on the learning rate of the inhibitory plasticity and the strength of excitatory synapses. In this section, we explore the impact of dynamic excitatory and inhibitory synapses on the ongoing activity, by systematically varying the same control parameters investigated in earlier studies on static networks, namely the external input rate ν_X_ and the inhibitory-excitatory balance *g*, which can be set via the ratio of absolute peak conductances γ as described in Section 2.1.1. In plastic networks *g* evolves throughout the simulation as a result of synaptic changes (see Supplementary Materials), so for ease of comparison with the static case we consider the network activity as a function of its initial value. Figure 2 shows the behavior of an example network as described in Section 2.1, with parameters set according to Section 2.3.1, leading to AI-type activity (Figure 2A). As desired and akin to its biological counterpart, the single neuron's spiking activity is highly irregular, with membrane potential hovering slightly below threshold (Figures 2B,F). Furthermore, the excitatory and inhibitory synaptic currents impinging onto this neuron are closely balanced (Figure 2C). This activity pattern is consistently conserved across the population, leading to the distributions observed in Figures 2D,E,G (these statistical descriptors were computed as described in Section 2.5.1). The synapses are subjected to modifications due to the ongoing activity and the stochastic input, leading to more narrowly distributed synaptic weights compared to the initial condition (Figure 2H). However, as the network maintains its AI-type activity during the evolution of synaptic strengths, the distributions of the statistical measures shown in Figures 2B–F remain essentially indistinguishable throughout the simulation period.

Our analysis in this section focuses on the most important statistical descriptors of population activity and on the generic computation capacity of the networks. In the first case, the measures are computed as described in Section 2.5.1 over a period of 20 s following a long initial equilibration phase. The network receives no specific input during this analysis, just the external input rate described above. Here, results are obtained from a single realization of each network configuration due to the computational intensity of the parameter scan. In the second case, the network receives independent Poissonian spike trains (as described in Section 2.5.2). Results are averaged over 10 realizations of each network configuration.

**Figure 3:**
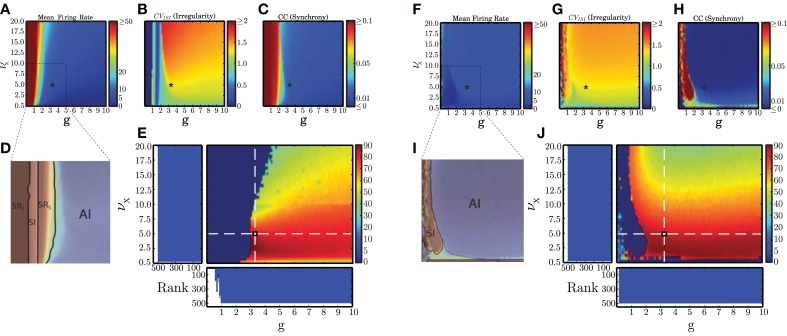
**Dynamical network states in static and plastic networks**. **(A–C)** Main properties of static network dynamics as functions of the control parameters ν_X_ (rate of external Poissonian drive) and *g* (effective excitation/inhibition balance): average firing rates, irregularity, and synchrony. **(D)** Schematic depiction of the different network states observed in static networks. This figure was obtained by overlaying **(B,C)** in the depicted range, which corresponds to the region where the most significant state transitions occur. **(E)** AI-score expressed as the percentage of neurons in the population that fire with a rate ≤20 spikes/s and whose CV_ISI_ ∈ [0.8, 1.5], in conditions where the average pairwise CC ≤ 0.05 (see Section 2.5.1). The histograms show the average results of the kernel quality analysis (Section 2.5.2, where Rank refers to the number of linearly separable columns of the state matrix in response to 500 different stimulus templates) along the two main axes (highlighted by the white dashed lines) over 10 analyses per condition. Note that the parameter combinations marked in **(A–C)** with a small star correspond to the point where these two main axes intersect. **(F–J)** As in **(A–E)** but for plastic networks.

The main results of our analysis over a broad parameter range are depicted in Figure 3 for static (**A–E**) and plastic (**F–J**) networks. The presence of plasticity strongly influences network activity. In accordance with the results presented in Kumar et al. (2008b), the static networks exhibited the asynchronous irregular (AI), fast and slow synchronous regular (SR_F_, SR_S_) and synchronous irregular (SI) regimes, but no asynchronous regular (AR) regimes were observed. In the plastic networks, only SI and AI regimes are observed, indicating that plasticity abolishes regular spiking activity except for a small region, where the external stimulus is weakest (ν_X_ = 0.5 spikes/s). Static networks with very weak inhibition (*g* < 1) have very high average firing rates, whereas plastic networks have low firing rates for almost all configurations. These results demonstrate that the presence of balanced plasticity makes the existence of the low rate AI dynamical state much more robust in comparison to static networks. The smooth profiles of the measures indicates that a single realization of the network configuration is sufficient to capture them.

We additionally measured the generic computation capacity of these networks, i.e., their ability to separate similar time-varying input streams in the form of fixed spike templates (see Section 2.5.2). Our results reveal that all regimes of the static network have a high generic computation capacity except SR_F_. This is demonstrated by the low rank in Figure 3E for network configurations in the SR regime identified in Figure 3D. In this regime, the dominant excitation and consequent excessive firing hinders a proper stimulus separation. For all other regimes, the rank is maximal, indicating that all the columns of the state matrix are linearly separable, allowing a fine discrimination of input stimuli. As plastic networks abolish the pathological SR regimes, every configuration of parameters leads to maximally separable circuit states (indicated by maximal ranks in Figure 3J), thus the presence of plasticity also increases the robustness of generic computation capacity in comparison to static networks.

Based on these results, we were able to select a suitable network configuration for our investigation of the capacity of static and plastic networks to extract information from structured input (described in Section 2.4), which comprises the main focus of our study. The selected configuration (marked with a star in all panels of Figure 3) produces activity with a high AI-score for both types of network. The parameters are ν_X_ = 5 spikes/s and *g* ≃ 0.29 γ, which for γ = 12 leads to *g* ≃ 3.479 (see also Section 2.3.1).

### 3.2. Stimulus discrimination

The ongoing network dynamics, when perturbed by an external stimulus pattern, performs a non-linear temporal expansion of its input, projecting it in a high-dimensional state-space as a complex, transient activity pattern (Rabinovich et al., 2008; Lukoševicius and Jaeger, 2009; Maass, 2010). In the following, we investigate whether balanced plasticity allows the network to counteract the effects of stimulation on the local E/I balance and develop stable stimulus representations, making the trajectories of network states more robust and easier to decode while maintaining suitable ongoing population activity.

#### 3.2.1. Effective discrimination with different input features

**Figure 4:**
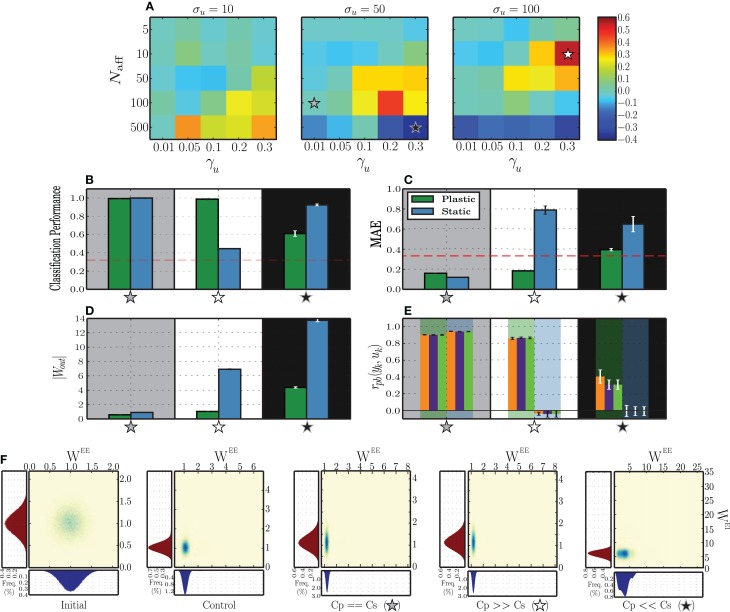
**Classification performance of readout neurons trained on the responses of static and plastic networks (*C*_s_, *C*_p_), obtained from 10 simulations per condition**. **(A)** Absolute difference in classification performance *C*_p_ − *C*_s_ as a function of peak input burst rate σ_*u*_, number of afferent neurons *N*_aff_ and proportion of total excitatory population receiving each input stimulus γ_*u*_. The stars mark three conditions of greater interest for further analysis, *C*_p_ ≫ *C*_s_ (white star; σ_*u*_ = 100, *N*_aff_ = 10, γ_*u*_ = 0.3), *C*_s_ ≫ *C*_p_ (black star; σ_*u*_ = 50, *N*_aff_ = 500, γ_*u*_ = 0.3), and *C*_s_ ≃ *C*_p_ (gray star; σ_*u*_ = 50, *N*_aff_ = 100, γ_*u*_ = 0.01). **(B–E)** Expanded results on the highlighted conditions, namely classification performance **(B)**, mean absolute error of the readout output **(C)**, vector norm of obtained readout weights **(D)** and point-biserial correlation coefficients between the readout output to each symbol (*y*_*k*_) and the corresponding binary target value (*u*_*k*_) (**E** each group of 3 bars corresponds to one network type (plastic or static), as highlighted by the background color). **(F)** Comparison of synaptic weight distributions for different conditions (from left to right): initial distributions, prior to any modification, control condition corresponding to the absence of patterned stimulation (only unspecific, background input (*X*)) and the three conditions of interest highlighted in the **(A–E)**. Note that the total range of values assumed by the synaptic weights in each condition is not easily discernible, but corresponds to the limits of the corresponding axes.

To better understand the dynamics underlying stimulus representation, we first analyse the absolute difference between static and plastic networks in terms of the performance obtained by readout neurons trained to classify the responses (Figure 4A). To do so, we use input sequences as described in Section 2.4, composed of *k* = 3 randomly ordered and sequentially presented stimuli.

The results show that plastic networks are not invariably better sources of classification information than static networks. When the peak rate of the input burst signals is low (σ_*u*_ = 10 spikes/s), the main differentiating factor is the number of afferent neurons that synapse onto each input population. Both static and plastic networks perform much better in the presence of a stronger input (*N*_aff_ ≥ 100) and when these input neurons connect to a larger sub-population. All other input parameters lead to insufficient discrimination, which is reflected in a readout classification performance at chance level for both network types (see Figure 4A and Supplementary Materials).

Increasing the input burst rate allows static networks to outperform plastic ones in conditions where the number of afferent neurons is high (*N*_aff_ = 500). Conversely, in conditions where the number of afferents is very low (*N*_aff_ = 5), the input is not strong enough to create a discernible response and both networks perform at a level barely above chance. This performance improves slightly as the number of receiving neurons increases. For intermediate values of afferent neurons, both networks display significantly discriminative responses, with the difference favoring mainly plastic networks, particularly if the size of the stimulated population is large (γ_*u*_ ≥ 0.1).

In the following sections, we carry out further analysis to uncover the reasons why in some cases plasticity increases the network performance and in other cases decreases it. In order to do this, we isolate three input conditions which lead to different comparative performances of the plastic and static networks. In one configuration, marked by a gray star in Figure 4A and examined in Section 3.2.2, plastic and static networks performed flawlessly. In the configuration marked by a white star, there is a clear and significant advantage of having plastic synapses. This is examined Section 3.2.3. Finally, in the configuration marked by a black star, plastic synapses confer a significant disadvantage, which we analyse in Section 3.2.4.

#### 3.2.2. Specialized population responses

We start by analysing the condition where both network types exhibited a high capacity to discriminate the stimulus patterns (configuration marked with a gray star in Figure 4A). Each input signal consists of a relatively large number of afferent neurons (*N*_aff_ = 100), whose peak rate is at an intermediate value (50 spikes/s) and whose target population is very concise, consisting of only 80 excitatory neurons (0.01 × *N*^E^). The stimulus representations developed by both network types are highly specific, allowing the readout to classify with near perfect accuracy and low error (Figures 4B,C). Furthermore, the solutions found by the regression algorithm are highly stable and accurately reflect the population activity (low |*W*^out^|, see Figure 4D) and each readout output *y*_*k*_ is highly correlated with its corresponding target *û*_*k*_ (Figure 4E).

**Figure 5:**
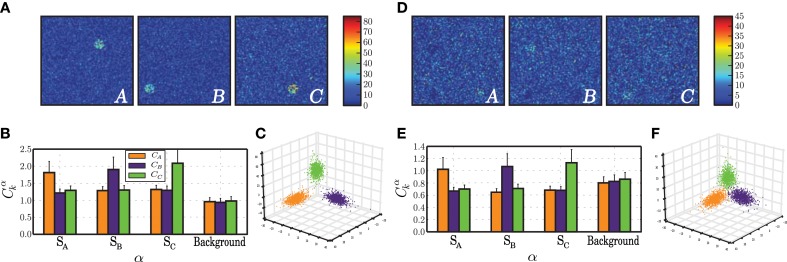
**Characteristics of population activity that allow a stable discrimination based on specialized population responses**. The results depicted in this figure correspond to the condition *C*_s_ ≃ *C*_p_ (σ_*u*_ = 50, *N*_aff_ = 100, γ_*u*_ = 0.01, gray star in Figure 4), obtained at the final stages of the stimulation period, i.e., after presenting the network with ≃ 1000 samples of each stimulus. **(A)** Snapshot of population activity represented as 2-dimensional firing rate maps, in response to each stimulus pattern (A, B, C) for a static network. The color of each point in the grid represents the average firing rate of the neuron located in the corresponding position, averaged over an interval spanning the stimulus duration plus the inter-stimulus interval. **(B)** Correlations between the firing rates recorded in each sub-population α = *S_A_*, *S_B_*, *S_C_*, Background of a static network in response to each signal *k*, with each of the input signals *s*_*k*_, averaged over 100 stimulus-response pairs. **(C)** Projection of network state vectors *x*_*u*_*k*__(*t*) in response to each stimulus pattern *u*_*k*_(*t*) onto the space spanned by the first 3 PCs. **(D–F)** as in **(A–C)** but for a plastic network.

A closer analysis of the network activity under these conditions provides a straightforward justification for the high discriminability of the responses. As can be seen in Figure 5A, upon receiving each stimulus pattern, the responsive sub-populations exhibit a clearly discernible activity that stands out from the background population, with a firing rate 30–40 spikes/s higher than that of the background, unstimulated neurons. This is less obvious in plastic networks, because the inhibitory plasticity rapidly counteracts the disruption of balance in the stimulated neurons, bringing their activity back to the background level within the time-course of a single stimulus presentation (Figure 5D). Due to this effect, the plastic network maintains low rates and an AI-score of 86 %, whereas the static network decreases to 69 % as a result of increased synchrony (data not shown).

In both networks, the strongly localized activity leads to highly specialized network responses whereby each sub-population's firing rates are highly correlated with that of their respective stimulus (Figures 5B,E). However, the correlation values are much lower in networks subjected to plasticity and so is the degree to which the population responses are specialized in relation to the background. The slightly degraded discriminability in plastic networks can also be seen by comparing the clustering of the circuit states in response to each pattern. Plastic network states cluster in well defined but less separated regions of state space than static network states (Figures 5C,F).

In summary, under input conditions where the stimulus has intermediate strength and the stimulated populations are very small, networks can easily produce a specialized response leading to accurate classification. The main effect of plasticity lies in its ability to maintain globally low average firing rates (approximately half of those displayed in the corresponding static case) and to ensure the stability and maintenance of the AI state.

#### 3.2.3. Plasticity stabilizes neural trajectories

**Figure 6:**
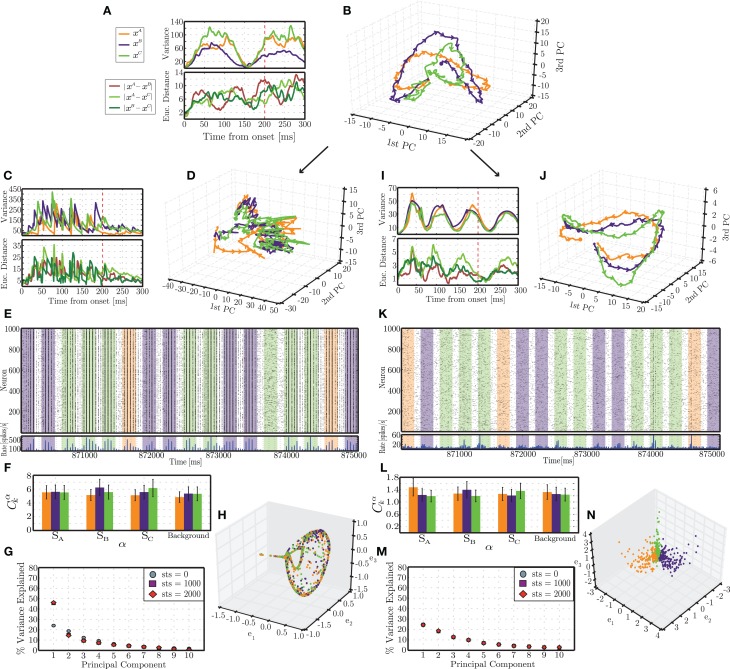
**Emergent trajectories of network states in response to each stimulus pattern in the initial stages of simulation (A,B) and following the presentation of 2900 stimulus patterns (i.e., 870 s of simulation), for static (C–G) and plastic (I–N) networks**. The colors used to highlight the responses to different stimuli are consistent with the previous figures (orange = *A*, blue = *B*, green = *C*). **(B,D,J)** Succession of network states in response to each stimulus, projected onto the space spanned by the first 3 PCs. Each trajectory reflects the mean of 10 responses recorded in different simulation periods and different conditions. **(A,C,I)** (top): Variance of each individual trajectory in relation to the mean; (bottom): average pairwise Euclidean distance between the depicted trajectories. **(E,K)** Snapshot of spiking activity of 1000 randomly selected excitatory neurons, recorded in the latest stages of simulation, over a period of 5 s. The shaded areas correspond to different stimuli. **(F,L)** Correlations between the firing rates of each population α with each input signal *s*_*k*_, averaged over 100 stimulus response pairs. **(G,M)** Amount of variance explained by the first 10 PCs throughout different phases of simulation (referred to as sequence time steps (sts)). **(H,N)** Representation of network states at the end of each stimulus pattern obtained by spectral embedding.

Several of the conditions depicted in Figure 4A resulted in a significant performance advantage for networks incorporating activity-dependent adaptation. To better elucidate the mechanisms underlying such advantage, we focus on the condition where the difference is most evident (highlighted with a white star in Figure 4) and analyse the dynamics of an individual network's responses to each stimulus pattern as they evolve along specific paths through the network's state space. It is worth noting that under the present input conditions (i.e., σ_*u*_ = 100, *N*_aff_ = 10, γ_*u*_ = 0.3), the responses are not discernible on the basis of a localized increase in firing rate among the stimulated neurons, which is reflected in the low degree of specialization of the population responses (Figures 6F,L). Hence, to understand the reasons underlying the performance difference, we must analyse the high-dimensional response dynamics to each stimulus, in the different network conditions.

Fully dissecting and understanding the dynamics of such high-dimensional dynamical systems is widely recognized as an extremely difficult, if not impossible task. We therefore resort to reduced-dimension descriptions and average measures that attempt to capture the essential phenomena as functions of a few variables expressing the most meaningful relations in the data. This has the advantage of allowing us to visualize the data and thus make some inferences and hypotheses about the underlying dynamics, but the disadvantage of providing a limited scope and ability to test the generality of these hypotheses in relation to the original state space.

The results depicted in Figure 6 reflect the activity of individual networks recorded at different points in time during the stimulation period. We will refer to these points as sequence time steps or *sts*. For each analyzed time point, the spiking activity in response to an individual stimulus is first low-pass filtered to create a response matrix containing the circuit states throughout the entire length of the response, until the onset of the subsequent stimulus. The dimensionality of each of these response matrices is then reduced by principal component analysis and their projections in the space spanned by the first three PCs analyzed. The procedure is repeated until 10 responses to each individual stimulus are obtained. We calculate the mean and variance of these responses to determine the stereotypy or variability of the transient activity patterns developed in response to the different stimuli starting from different network conditions.

In the first few sequence time steps (starting from *sts* = 0), the network responses already show a certain degree of stereotypy and the trajectories progress through distinct, albeit overlapping, regions of state space (Figure 6B). The average pairwise distances between trajectories show no specific pattern other than an increasing trend (Figure 6A, bottom). A striking feature, which we will come back to later, is the existence of a clear pattern in the variance of the trajectories. These initial results are remarkably similar among the different conditions (static and plastic networks) as well as among different random network instantiations, reflecting the initially similar embedding state space, obtained by tuning the ongoing, background activity dynamics. The trajectories are not exactly the same but tend to occupy similar regions of space and display a very similar pattern of variances.

After being presented with a long sequence of stimuli, the response patterns differ dramatically between the static and the plastic conditions. These results are depicted in the bottom part of Figure 6 (**C–H**, for static networks and **I–N** for plastic networks), and were obtained from *sts* = 2900. The trajectories of network states observed in static networks are now highly variable (with a variance about 4 times larger than in the initial steps; Figure 6C, top) and the different stimulus responses clump together, hampering an adequate discrimination (Figure 6D). In contrast, the trajectories observed in plastic networks have become more stereotypical, with a maximum variance approximately half of that verified in the initial condition (Figure 6I, top) and the responses become more “organized,” consistently unfolding throughout specific paths (Figure 6J).

Furthermore, the dimensionality of the response dynamics is also significantly different which has an obvious impact on their linear separability. The dimensionality of the state-space can be inferred by the amount of total variance explained by successive dimensions obtained by PCA. Figure 6G shows that, in static networks, after *sts* = 1000, the first PC accounts for just under 50% of the variance, compared to ~25% at *sts* = 0, which stands in clear contrast with the dynamics of plastic networks, where the percentage of explained variance remains invariant along the full stimulation time (Figure 6M). The low dimensionality and low separability of the static network's responses is further demonstrated in the result displayed in Figure 6H, which was obtained by spectral decomposition of the matrix of Euclidean distances between the state vectors (network states at *t*_0_ = 200 ms, see the dimensionality reduction section of Section 2.5.3). This figure depicts the existence of a low-dimensional manifold, where all the states in response to the different input patterns lie.

Conversely, networks that have been shaped by plasticity learn to explore the state space much more effectively, partly by virtue of the maintenance of the AI-type dynamics (Figure 6K), which supplies the network with a higher dimensional space over which to develop its responses (Figures 6M,N), in contrast with the static network where the activity tends to become more synchronized (Figure 6E), thereby increasing the redundancy of the individual neuron's responses resulting in a consequent reduction in dimensionality.

The variance of the analyzed responses also shows, at this stage, a clear periodic pattern where, at relatively constant intervals (≃ 70 ms), the 10 individual trajectories converge (see Figure 6I). In the initial state (*sts* = 0), the variances already showed a similar pattern, with a convergence point at *t* ≃ 150 ms after stimulus onset (Figure 6A). This is an interesting and somewhat unexpected result. Since these points do not reflect any meaningful property of the stimulus, they must reflect properties of the response dynamics that the network develops. We hypothesize that these points represent spontaneously generated saddle nodes that stabilize the dynamics along the unfolding trajectories, improving robustness and reproducibility. As the different trajectories approach these regions of state-space they are attracted to these points (hence the observed reduction of variance) and, after leaving these regions, the trajectories are repelled and allowed to diverge until the next saddle node captures them.

An additional question that arises from these results is whether the increased discriminability of the population responses in the plastic networks can be accounted for by the macroscopic dynamical state of the network (i.e., the ability to maintain a stable AI activity pattern both for ongoing and stimulus-driven activity) or whether the fine details of the learned synaptic connections are strictly necessary. To address this question, we perform two simple experiments, described in the following.

As discussed in Section 3.1, the effect of plasticity modifies the effective balance *g*, leading to a final value that is much larger than the initial one (see Supplementary Materials). Therefore, as a result of learning, the network will be strongly dominated by inhibition and placed in a dynamic regime where ongoing activity is more strongly of the AI type (see Figure 3). To determine whether the macroscopic dynamical state is sufficient to account for the network performance, we investigate a static network initialized with *g* ≃ 12 (similar to the final value of *g* obtained in the plastic network). The readout classification performance of this strongly inhibitory network shows a considerable improvement over the more weakly inhibitory network considered in this section (*C*_s_ ≃ 0.9667 as compared to *C*_s_ ≃ 0.445), thus reducing the performance difference from 0.54 to 0.02.

This result seems to support the first hypothesis, i.e., that the increased discriminability is due solely to the network's dynamical state. However, a second experiment suggests that this view is too simplistic. We analyse the classification performance obtained if the learned synaptic weights are randomly shuffled, losing any relevant structure. To do so, 3000 stimulus samples are presented to the plastic network, after which its synaptic weights are frozen and plasticity disabled. Subsequently, the recorded weights are randomly shuffled among the existing synapses and the network is exposed to a new sequence of 3000 stimulus samples. The responses to this second set of stimuli is recorded and used to train and test the readout's classification performance (following the same procedure described in Section 2.5.3). In this situation, the classification performance drops to chance level (*C*_p_ ≃ 0.33148).

Based on these results, we can conclude that the macroscopic dynamical state of the network is critical to achieve a high stimulus discrimination and consequent readout performance. For that reason, a random network can achieve very high performance if its initial state is placed in a strong AI regime. However, if the network connectivity is not random, but pre-structured by Hebbian learning in response to the training data, the fine details of connectivity that arise from the learning process play a key role in the maintenance of adequate stimulus representations; randomly re-organizing this connectivity structure results in a drop in performance to chance level. So, in this situation, the results do not rely exclusively on the global E/I balance (which is maintained after shuffling), but also require the conservation of the pre-learned weight structure. These phenomena are obviously not independent as the learned connectivity structure emerges to counteract the disruption of balance and to stabilize the activity in the AI regime. Randomly shuffling the synaptic weights may result, for example, in a decreased inhibition toward certain stimulated neurons, that consequently fire excessively and thus destabilize the global network dynamics. Indeed, the activity in the shuffled condition displays a higher amount of synchronous population activity (data not shown).

#### 3.2.4. Strong stimulation hinders representation

In some cases, the presence of plasticity reduced the network's ability to represent the input into distinct activity patterns, e.g., the configuration marked with a black star in Figure 4A. The conditions that allow this to occur are characterized by intermediate or high peak firing rate and high number of afferents, i.e., very strong input. However, note that although the classification performance is higher for the static network (Figure 4B), all other metrics show the reverse effect. The absolute error of the readout output is higher for static networks (Figure 4C) and the solutions found by the regression algorithm for the output weights are quite unstable, relying heavily on some state variables in detriment of others (Figure 4D). This means that only a certain fraction of the population effectively communicates the relevant information to the readout. Even then, the output does not provide a good match to the target binary values, a result that is further reinforced by the point-biserial correlation between the readout output and the target output, which is close to or below 0 (Figure 4E).

**Figure 7:**
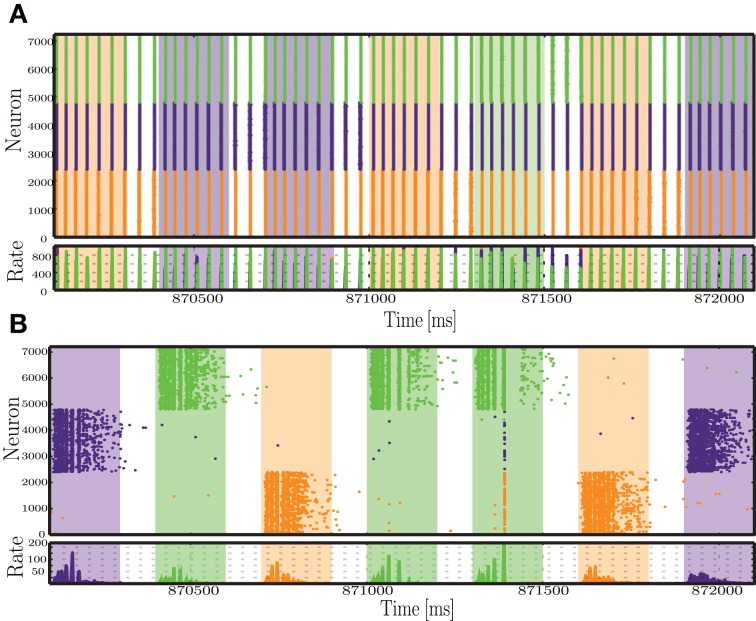
**Snapshot of the activity recorded within each of the stimulated sub-populations, during a period of 2 s, at the final sequence time steps (*sts* = 2900-2907), for static **(A)** and plastic **(B)** networks, in the condition where *C*_s_ >> *C*_p_**.

Examining the network activity in these conditions provides an idea to the mechanisms underlying these results (Figure 7). In plastic networks, the input is too strong and causes the inhibitory synapses to become excessively strong to counteract the equally excessive excitatory drive. The result is an almost completely silenced excitatory population, where the only sparse spiking activity appears as short-lived bursts in immediate response to each input. On the other hand, static networks also develop an unfavorable dynamic state, where most of the activity is punctuated by synchronous, population-wide bursts. The stimulated neurons are briefly and slightly decoupled from the burst, which allows some separation of the responses. The readout algorithm captures mostly the activity of the input populations and heavily amplifies the weights from these neurons. This then leads to an output sequence that, despite a correct label assignment (i.e., the largest output values at each time step are assigned to the correct symbol), consists of disproportionately large values, which justify the large absolute error and the low correlations (Figures 4C–E).

### 3.3. Differential effects of plasticity

The results presented so far show that the action of plasticity modulates the network's ongoing activity, endowing it with the ability to maintain Asynchronous Irregular states over a broader range of parameters and abolishing pathological states of Synchronous Regular activity (which we demonstrate to result in an impaired computational performance), when driven by a constant, stochastic and unspecific input (Section 3.1). In addition, as discussed in Section 3.2, if sequentially structured and topographically mapped input patterns are introduced, their interaction with the ongoing activity and the manner in which they disrupt local E/I balance (namely the strength and the spread of the disruption) determines the ability of networks operating in inhibition-dominated regimes to adopt adequate representations, i.e., to utilize bounded stimulus-specific sub-spaces. Different combinations of input features are shown to be able to cause discernible (linearly separable) population responses, regardless of the presence of adaptation. However, the characteristics of the adopted responses demonstrate that the action of plasticity is strictly necessary to maintain a suitable, “healthy” population activity by avoiding the pathological Synchronous Regular regimes toward which static networks are driven in the presence of strong stimulation (Section 3.2.4).

In the previous experiments we did not consider how the two different types of plasticity interact. We now turn our attention to disentangling the roles of the different plasticity mechanisms under study to determine whether the improvements observed in the development of stimulus representations are the product of a combined, synergistic action of these mechanisms, whether one of them plays a dominant role or whether they even counteract each other's effects. As we demonstrate in Figure 4F (see also Supplementary Materials), the steady-state weight distributions in the different conditions (disregarding the pathological states observed in the condition *C*_s_ ≫ *C*_p_ Section 3.2.4) do not differ noticeably from those developed in a control condition when no patterned stimuli are delivered, and consequently are not informative about these differential effects of eSTDP and iSTDP. We therefore adopt the configuration of input parameters that leads to the greatest performance of plastic networks with respect to static networks (*C*_p_ ≫ *C*_s_, condition marked with a white star in Figure 4: σ_*u*_ = 100, *N*_aff_ = 10, γ_*u*_ = 0.3), and assess their performance in situations where the network dynamics is shaped by neither (Static), one (iSTDP/eSTDP) or both (Plastic) of the plasticity mechanisms. We systematically vary the task difficulty by building stimulus sequences with an increasing number of stimuli (*k*) thus requiring a matching number of discernible network responses in order to be discriminable.

**Figure 8:**
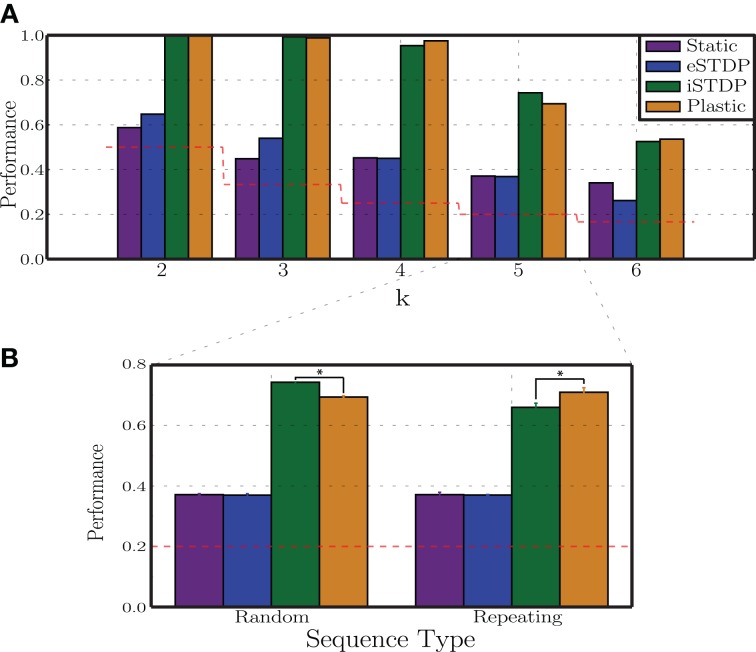
**Differential impact of plasticity mechanisms on classification performance**. **(A)** Readout classification performances as a function of sequence complexity (variable *k*), for networks with no (static), one (eSTDP/iSTDP) or both (plastic) plasticity mechanisms. Depicted results refer to a single realization per condition. **(B)** Performance in classifying 5 different stimuli in conditions in which the sequences are randomly ordered or present a repeating pattern. Depicted are the means and standard deviations over 10 simulations per condition (^*^p < 0.005).

The results of this analysis are depicted in Figure 8A. The most striking result is the clear dominance of iSTDP, which is solely responsible for most of the observed performance improvement in relation to the static condition. Working alone, eSTDP is only marginally advantageous in the less demanding task conditions (*k* = 2, 3) and, as the task difficulty increases, its actions result in no net improvements. In the extreme case, the presence of eSTDP can even undermine the network's representational abilities and decrease the overall performance to a level barely above chance (when *k* = 6). On the other hand, iSTDP alone accounts for the majority of the observed results; the addition of eSTDP can even decrease the readout performance (*k* = 5). In most cases, plastic networks with both mechanisms active perform as well as iSTDP alone or worse.

These results demonstrate that the main feature responsible for the increased discriminability of stimulus-driven population responses in plastic networks is the decorrelating action of iSTDP and the consequent maintenance of the AI dynamic regime both for ongoing activity and stimulus-driven responses. These findings contradict our initial hypothesis that a synchronous burst of activity impinging on particular groups of neurons would bind the neurons belonging to these clusters by introducing correlations in their driven activities, and by so doing aid the discrimination of network states. However, no such clusters formed (see Supplementary Materials), due to the decorrelating action of iSTDP which hinders the efficacy of correlation-driven eSTDP.

The Hebbian nature of eSTDP is well suited to uncover causal relations in the input structure, which prompts the question of whether it would have a more beneficial effect on discriminability if the input contained clear causal relations rather than randomly drawn stimuli (see Section 2.4). We therefore performed an additional test in which the sequences were composed of *k* = 5 stimuli arranged in a fixed pattern, repeated throughout the entire stimulation period. The results of this analysis (shown in Figure 8B) demonstrate that, while eSTDP alone does not perform any better on the repetitive pattern (when compared with the random pattern), plastic networks with both eSTDP and iSTDP active perform somewhat better than iSTDP alone. So, even in the presence of a clear causal structure in the input data, the effect of eSTDP seems to be negligible which is an intriguing result. However, it should be noted that, while these results raise some questions, they are not fine-grained enough to allow us to draw broader conclusions. We note, for example, that the inter-stimulus interval (100 ms) used in these experiments is much longer than the relevant time window for eSTDP modifications (20 ms), thus diminishing its ability to learn the causal structure of the sequential input events. Further studies are strictly necessary to clarify some of these results and address the issues they raise.

## 4. Discussion

A primary function of neocortical circuits lies in their ability to dynamically adopt and sustain reliable representations of sequentially occurring perceptual events in a self-organized and experience-dependent manner (Brosch and Schreiner, 2000; Zacks et al., 2007; Rabinovich et al., 2008; Buonomano and Maass, 2009). They need to maintain the necessary flexibility to adequately respond to sudden transitions that may require a global shift in representational space, while retaining a certain amount of contextual information. These characteristics are necessary for any further processing to occur (such as the dynamic evaluation of sequential dependencies present in the input), however, they entail an apparent contradiction between sensitivity and robustness. It seems probable that this is resolved via functional remodeling and adaptation, involving modifications at different spatial and temporal scales mediated by a combination of different synaptic and intrinsic mechanisms.

In this study, we have explored the relations between several important organizational principles of functional neurodynamics, involving distributed processing in inhibition dominated, sparsely coupled recurrent networks, whose rich ongoing dynamics supports the emergence of stimulus-specific spatiotemporal activity patterns. We have shown that the action of dynamic excitatory and inhibitory synapses, modulated by spike timing-dependent mechanisms, has a significant impact on the robustness and active maintenance of an ongoing activity state characterized by irregular firing that is asynchronous across the network. In this *asynchronous irregular* regime, the network activity is considered to most closely resemble cortical spiking activity *in vivo* (Vaadia and Aertsen, 1992; Softky and Koch, 1993; Shadlen and Newsome, 1994; Brunel, 2000; Destexhe, 2009; Ostojic, 2014).

We have additionally established an objective relation between the dynamic states of ongoing activity (characterized by varying degrees of synchrony and regularity) and generic online processing capacity, demonstrating that pathological network-wide synchronization observed in the synchronous regular regime hinders the ability to properly map spatiotemporal input streams into discernible activity states, a process necessary for online computation on time-varying inputs. By abolishing such dynamic regimes (see Figure 3), balanced plasticity increases the robustness of generic computational capacity, thus expanding the efficacy of these circuits as information processing devices.

The sequential interaction of spatiotemporal input patterns with the ongoing network activity modifies the dynamics of the stimulated neurons and, via waves of recurrent interactions, also that of the global network on which they are embedded. These modifications are highly heterogeneous, depending on the nature and characteristics of the input stimulus. Experimental evidence shows that increased thalamic input is related to a higher degree of asynchronous activity in sensory cortices (Cohen and Maunsell, 2009; Poulet et al., 2012; Tan et al., 2014), which emphasizes the relevance of AI-type activity both as the ground state (Shadlen and Newsome, 1994, 1998; Vogels et al., 2005) and the active state of cortical activity, even though these two states may be characterized by different statistical features (Ostojic, 2014). In order to ascertain how certain features of the stimulus influence the network responses and modify the observed dynamics, we have driven the networks with specific input stimulus “events,” characterized by spike bursts of different amplitudes (as depicted in Figure 1), mimicking the thalamic burst mode of firing (Ramcharan et al., 2000; Sherman, 2001b; Bruno and Sakmann, 2006). These events impinge on a variable number of afferent neurons and target topographically arranged (Thivierge and Marcus, 2007; Silver and Kastner, 2009) subsets of excitatory neurons, thus momentarily disrupting the local E/I balance. The objective was to assess the quality and characteristics of dynamic stimulus representations developed by networks whose synapses are endowed with plasticity, enabling them to counteract the local disturbances, with networks whose synapses are fixed and static, in relation to the strength and spatial distribution of the stimuli.

Our results demonstrate that, in input conditions where the stimulus has intermediate strength but the stimulated populations are very small and spatially concise, the main effect of plasticity lies in its ability to maintain globally low average firing rates (approximately half of those displayed in the corresponding static case) and to ensure the stability and maintenance of the AI state (Figure 5). On the other hand, if the input is too strong, comprising the activity of a large number of afferent fibers, activity becomes highly pathological, even if plasticity is present. Whereas plastic networks become largely silent due to excessive inhibition that emerges to counteract the equally excessive excitatory drive, static networks become highly synchronized and fire in short population bursts (see Figure 7).

However, strong and highly focussed stimuli are probably not representative of typical cortical input. We also considered scenarios where the stimulus was weaker and the receiving populations were large and distributed enough to avoid a strong localized response. In these situations, plasticity is shown to be generally beneficial (although not universally so), by allowing the network to efficiently explore a higher dimensional state space, achieved via the maintenance of AI-type activity (Figures 6K–N). The reduction in the dimensionality of the dynamic state observed in static networks, on the other hand, is a signature of an increasingly constrained and redundant dynamical space, which is detrimental to an adequate stimulus representation (Figures 6D–H). Plasticity is also shown to improve robustness and stereotypy of the successions of network states developed in response to each stimulus pattern (Figures 6I,J). Such transient, but trial-to-trial reproducible sequences of neural activity have been demonstrated experimentally in several sensory systems (e.g., Brosch and Schreiner, 2000; Mazor and Laurent, 2005; Broome et al., 2006; Rabinovich et al., 2008) and play a critical role in neural computation.

The pattern observed in the response trajectories demonstrated the existence of regions of negligible variance along the system's response trajectories. We hypothesize these regions to represent saddle nodes, i.e., metastable states, whose temporal order and location is determined by the network's self-organized functional connectivity. This hypothesis is consistent with known principles of neurodynamics (Rabinovich et al., 2006), namely the formation of stable heteroclinic sequences (Rabinovich et al., 2008; Rabinovich and Varona, 2011). The transformation of incoming stimuli into the spatiotemporal activity of a neuronal ensemble is represented as a heteroclinic sequence made up of many saddle nodes, and heteroclinic orbits connecting them, and whose specific architecture is stimulus-dependent and reproducible. Plasticity increases the number of such points along each stimulus representation—from Figure 6A (top) to Figure 6I (top), the number of low variance points along the response grows from one to three. This finding is interesting, as it suggests that activity-dependent self-organization adjusts the network dynamics in a manner that improves the resilience and reproducibility of each response to a specific stimulus, while maintaining an adequate underlying dynamics that keeps the network sensitive to external modulations.

Obviously, caution is warranted in relation to this interpretation of the data—since we are discussing the dynamics observed in a low-dimensional projection space, no definitive or absolute conclusions may be drawn regarding the original state space. However, the reproducibility of this pattern of results over a range of different random network instantiations and different initial conditions provides some support for the hypothesis. Furthermore, the formation of this stable periodic pattern of variance is only visible after a long training period. Analysing intermediate time points shows a gradual transition, where the number and frequency of low-variance regions varies among stimulus responses (data not shown). Nevertheless, further analysis is necessary to validate this argument. It would be interesting to obtain a low-dimensional formulation of the network dynamics under these conditions and carefully explore it to gain a better insight into the underlying mechanisms. This could be done, for instance, by eigenfunction expansion, which could provide a reasonable approximate low-dimensional dynamical system that would allow careful analytic treatment.

Additional expansions of the current work could involve the use of different input stimuli, combinations of stimuli or the inclusion of temporal dependencies between sequence elements. Most of the input-dependent results we have analyzed (with the exception of Section 3.3), although involving stimulus sequences, are based solely on stimulus discrimination and representation given that the stimuli are randomly ordered. Under the theory of stable heteroclinic sequences, we would expect that plasticity would allow the network to develop sequence representations, where each element would be dynamically represented by its own saddle node, and full sequence memory would be encoded by a transient motion in state space along the paths specified by these metastable states. It would also be interesting to investigate whether the capacity of the networks to maintain an AI regime when perturbed would allow them to perform balanced amplification of specific activity states, as has recently been demonstrated for networks incorporating optimally tuned inhibition (Hennequin et al., 2014).

In summary, the most relevant conclusion to draw from the current results is that the quality of dynamical representations adopted in response to sequential stimulus patterns is very much dependent on the maintenance of the AI-type activity, which not only provides a stable high-dimensional embedding manifold (in the form of ongoing activity) from which stimulus-specific responses arise, but also shapes the stability and robustness of those responses, that must evolve through bounded trajectories through the network's state-space. In the present study, the ability to maintain these regimes in the face of variable disruptions was achieved by the decorrelating action of iSTDP, which accounts for the results displayed in Figure 8A. As the precise difference between spike times is not strictly required for this, it is reasonable to assume that simpler synaptic or intrinsic mechanisms may be equally capable of stabilizing the network's dynamics allowing it to support equally rich dynamical stimulus representations.

These results also raise important and intriguing questions. Given the limited role played by eSTDP in our study, what is its true functional relevance? Some argue that either the functional relevance of eSTDP in the adult cortex has been overstated (Lisman and Spruston, 2005, 2010) or that it must rely on more complex intracellular mechanisms, that are not fully captured by the current formulations (Shouval et al., 2010; Shulz and Jacob, 2010), which are largely based on *in vitro* recordings. We are not in a position to provide definitive answers to address this question, but given that properly representing stimulus events as they unfold over time is a necessary first step toward more complex computations, our demonstration that this ability does not require eSTDP, but relies on the homeostatic process of regulating ongoing activity by active decorrelation, provides some interesting material to this debate and opens up a new set of questions.

Which mechanisms account for the brain's ability to represent stimulus events occurring over variable time scales (most of which much longer than those relevant for STDP modifications) and discover causal relations between them? These are fundamental steps in most cognitive processes, and must rely on some degree of lasting functional modifications. These processes are likely to rely on a complex interplay of various sub-processes, of which eSTDP and iSTDP may be an integral part of. The pressing need to address this type of questions, spanning multiple spatiotemporal descriptive scales reinforces the relevance of studies involving a synergistic combination of multiple adaptation mechanisms.

## Funding

Partially funded by the Erasmus Mundus Joint Doctoral Program EuroSPIN, BMBF Grant 01GQ0420 to BCCN Freiburg, the Junior Professor Program of Baden-Württemberg, the Helmholtz Alliance on Systems Biology (Germany), the Initiative and Networking Fund of the Helmholtz Association and the Helmholtz Portfolio theme Supercomputing and Modeling for the Human Brain.

### Conflict of interest statement

The authors declare that the research was conducted in the absence of any commercial or financial relationships that could be construed as a potential conflict of interest.
