# Supplementary material for: Dynamic stability of sequential stimulus representations in adapting neuronal networks
Source: Front Comput Neurosci. 2014 Oct 22;8:124. doi: 10.3389/fncom.2014.00124 (PMC4205815; doi:10.3389/fncom.2014.00124)
Supplement: Supplementary file 1 [file Presentation1.PDF]

Frontiers in Computational Neuroscience

Research Article
September 26, 2014
10.3389/fncom.2014.00124

Supplementary Material: Dynamic stability of sequential stimulus representations in adapting neuronal networks

Renato Duarte [1,2,3,4*] **and Abigail Morrison** [1,2,3,5]

[1] *Institute of Neuroscience and Medicine (INM-6) and Institute for Advanced Simulation (IAS-6), Jülich Research Center and JARA, Jülich, Germany*
[2] *Bernstein Center Freiburg, Albert-Ludwig University of Freiburg, Freiburg im Breisgau, Germany*
[3] *Faculty of Biology, Albert-Ludwig University of Freiburg, Freiburg im Breisgau, Germany*
[4] *Institute of Adaptive and Neural Computation, School of Informatics, University of Edinburgh, United Kingdom*
[5] *Institute of Cognitive Neuroscience, Faculty of Psychology, Ruhr-University Bochum, Bochum, Germany*

Correspondence*:
Renato Duarte
Jülich Research Center and JARA,
Institute of Neuroscience and Medicine (INM-6) and Institute for Advanced Simulation (IAS-6),
Building 15.22, 52425 Jülich, Germany, r.duarte@fz-juelich.de

Emergent neural computation from the interaction of different forms of plasticity

SUPPLEMENTARY FIGURES

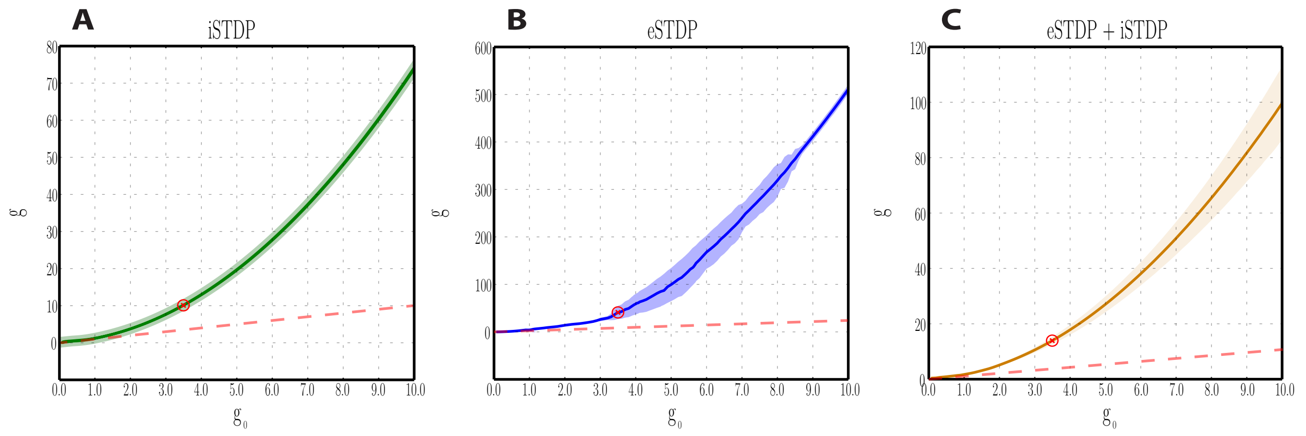

Supplementary Figure 1. Evolution of the E/I balance in networks subjected to the effects of the different plasticity mechanisms. The results displayed refer to the mean and standard deviation of the g parameter (see main text), over the different values of ν_X tested, obtained at the end of each simulation and plotted as a function of the initial value of g (referred here as g_0), for networks with only inhibitory STDP active (**A**), only excitatory STDP (**B**), or both (**C**). The point highlighted in all 3 curves corresponds to $g_0 = 3.479$, which marks the selected parameter used to investigate the capacity of the analysed networks to extract information from structured input (see main text, Sec. 3.2).

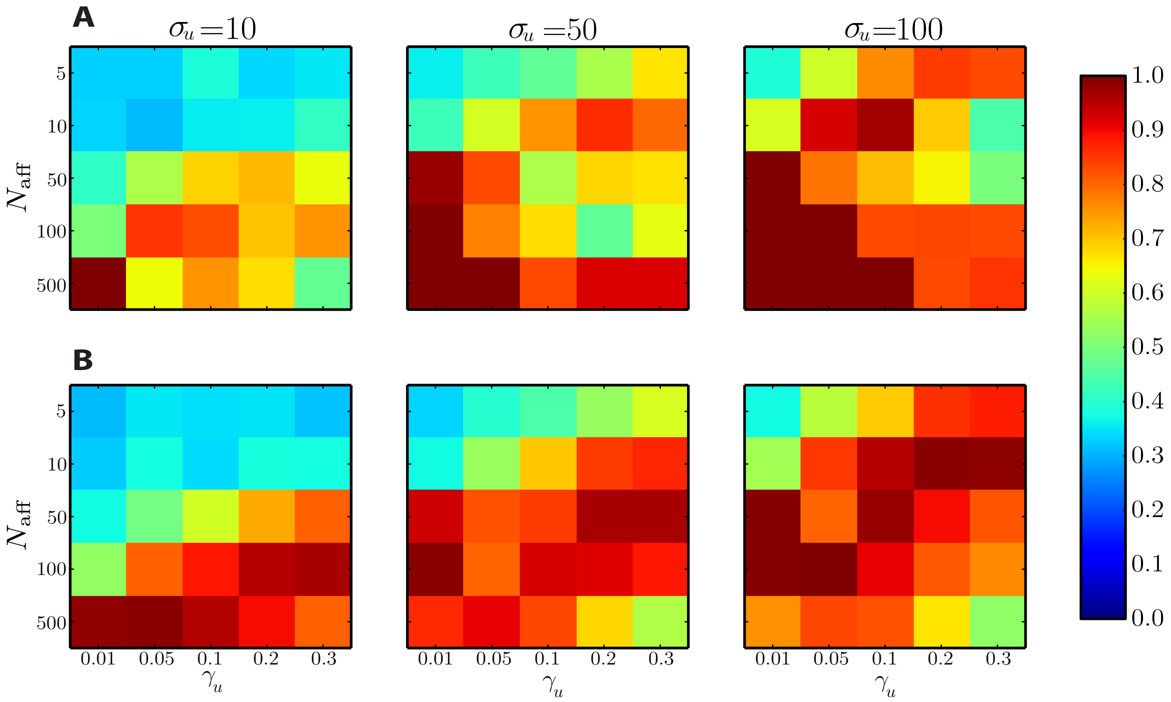

Supplementary Figure 2. Performance values obtained by readout neurons trained to classify the network responses to 3 different stimulus patterns, as a function of the input parameters, for static (C_{s}, **A**) and plastic (C_{p}, **B**) networks. The results depicted in Fig. $4A$ in the main text correspond to subtracting the panels in **A** from the corresponding panels in **B**, i.e. $C_{\mathrm{p}} - C_{\mathrm{s}}$.

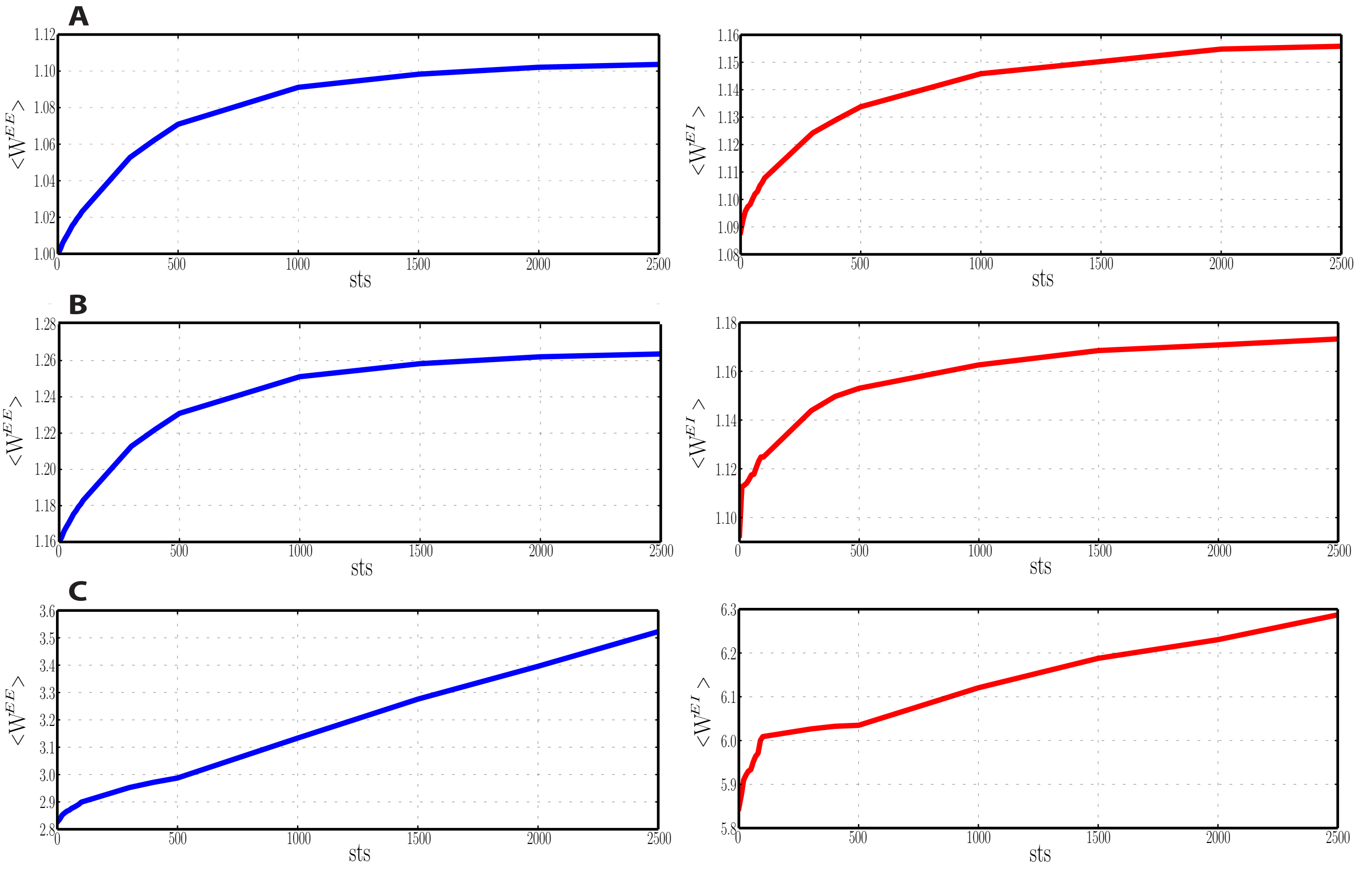

Supplementary Figure 3. Evolution of the mean synaptic weights in the 3 main conditions analysed over the course of 2500 simulation time steps (sts), after discarding the initial 300 steps. The panels on the right (blue) refer to the excitatory weights, w^{EE} whereas the panels on the left refer to the inhibitory weights w^{EI}. **A**: Condition where $C_{\mathrm{s}} \simeq C_{\mathrm{p}}$ (gray star marker in Fig. 4; $\sigma_{\mathrm{u}} = 50$, $N_{\mathrm{aff}} = 100$, $\gamma_{\mathrm{u}} = 0.01$). **B**: $C_{\mathrm{p}} \gg C_{\mathrm{s}}$ (white star marker in Fig. 4; $\sigma_{\mathrm{u}} = 100$, $N_{\mathrm{aff}} = 10$, $\gamma_{\mathrm{u}} = 0.3$). **C**: $C_{\mathrm{s}} \gg C_{\mathrm{p}}$ (black star marker in Fig. 4; $\sigma_{\mathrm{u}} = 50$, $N_{\mathrm{aff}} = 500$, $\gamma_{\mathrm{u}} = 0.3$). In the conditions depicted in **A** and **B** the weights reach a steady-state towards the end of the considered simulation period. This is, however, not the case for the pathological condition depicted in **C**.

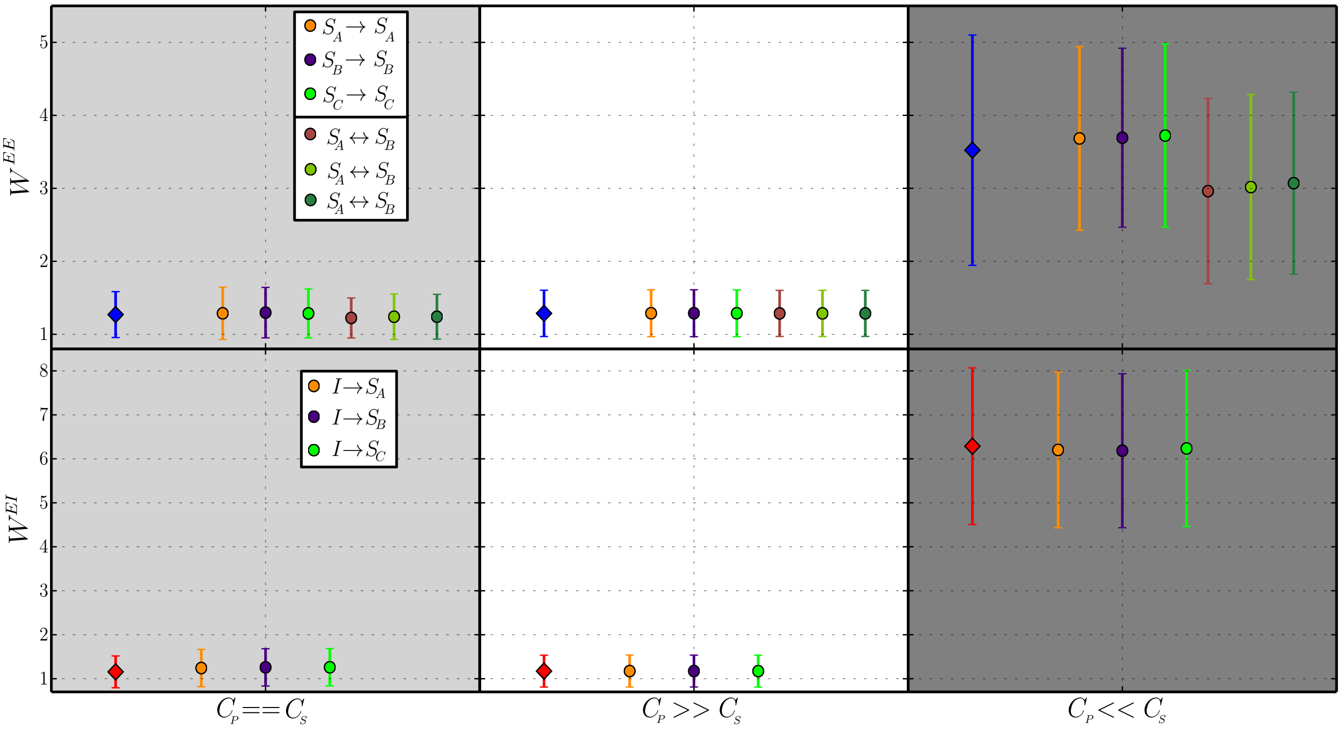

Supplementary Figure 4. Mean and standard deviation of the weight distributions involving the stimulated neuronal sub-populations for the 3 main conditions analysed. The top panel refers to the excitatory synaptic weights within and between the stimulated sub-populations, whereas the bottom panel refers to the inhibitory synaptic weights impinging onto each of the sub-population's neurons. All conditions are compared with the features of the weight distributions over the entire population (marked in each panel by the blue (EE synapses) and red (EI synapses) diamond markers).

SUPPLEMENTARY TABLES

Supplementary Table 1. Tabular description of network model after **Nordlie et al.** (2009)

**A: Model Summary**	
**Populations**	Three main: excitatory (E), inhibitory (I), external (X); Variable input-specific sub-populations (S_k) ($\subset$ E, if applicable)
**Topology**	Regular 2D grid lattice with periodic boundary conditions
**Connectivity**	Random Recurrent Connections
**Neuron model**	Leaky integrate-and-fire, fixed voltage threshold, fixed absolute refractory time
**Synapse model**	Conductance-based, exponentially decaying PSCs
**Plasticity**	eSTDP in excitatory to excitatory connections, iSTDP in inhibitory to excitatory connections
**Input**	Independent fixed-rate Poisson spike trains to all neurons (background), independent variable rate Poisson spike trains (burst signal) delivered to specific subpopulations
**Measurements**	Spiking activity

**B: Populations**		
**Name**	**Elements**	**Size**
Global E	IaF neuron	$N^{\mathrm{E}} = 4N^{\mathrm{I}}$
Global I	IaF neuron	N^{I}
Input-Specific	IaF neuron	$N_{\mathrm{u}} = \gamma_{\mathrm{u}} \times N^{\mathrm{E}}$

**C: Connectivity**			
**Name**	**Source**	**Target**	**Pattern**
EE	E_j	E_i	Random, ϵ density, plastic weight $\bar{g}^{\mathrm{E}} w_{ij}^{\mathrm{EE}}$, delay d
EI	I_j	E_i	Random, ϵ density, plastic weight $\bar{g}^{\mathrm{I}} w_{ij}^{\mathrm{EI}}$, delay d
IE	E_j	I_i	Random, ϵ density, fixed weight $\bar{g}^{\mathrm{E}} w^{\mathrm{IE}}$, delay d
II	I_j	I_i	Random, ϵ density, fixed weight $\bar{g}^{\mathrm{I}} w^{\mathrm{II}}$, delay d

**D: Neuron Models**	
**Name**	Iaf neuron
**Subthreshold dynamics**	if $(t > t^* + \tau_{\mathrm{ref}})$<br>$C_{\mathrm{m}} \frac{dV_i(t)}{dt} = g_{\mathrm{leak}} \left(V_{\mathrm{rest}} - V_i(t)\right) + I_i^{\alpha \mathrm{E}}(t) + I_i^{\alpha \mathrm{I}}(t) + I_i^{\alpha \mathrm{X}}(t)$<br>else<br>$V(t) = V_{\mathrm{reset}}$<br>$I_{ij}(t) = g_{ij}(t)(V_\beta - V_i(t))$
**Spiking**	If $V(t-) < \Theta$ OR $V(t+) \geq \Theta$<br>1. set $t^* = t$ 2. emit spike with time stamp t^*

**E: Synapse Models**	
**Synaptic trace**	$\frac{dx_i(t)}{dt} = -\frac{x_i}{\tau_{\mathrm{p}}} + \sum_{t_i} \delta(t - t_i^*)$
**Name**	Excitatory STDP (eSTDP) (**van Rossum et al.** (2000))
**Type**	'Hybrid' update rule, additive for potentiation, multiplicative for depression
**Spike pairing scheme**	All-to-all (**Morrison et al.** (2008))
**Pair-based update rule**	$\Delta w_+^{\mathrm{EE}} = \lambda \exp(-\lvert\Delta t\rvert/\tau_{\mathrm{p}})$<br>$\Delta w_-^{\mathrm{EE}} = \alpha_{\mathrm{ep}} \lambda w^{\mathrm{EE}} \exp(-\lvert\Delta t\rvert/\tau_{\mathrm{p}})$ synaptic delay considered to be 100% dendritic
**Online update rule**	$\frac{dw_{ij}^{\mathrm{EE}}}{dt} = \alpha_{\mathrm{ep}} \lambda w^{\mathrm{EE}} x_i(t) \delta(t - t_j^*) + \lambda x_j(t) \delta(t - t_i^*)$
**Name**	Inhibitory STDP (iSTDP) (**Vogels et al.** (2011))
**Type**	Symmetric update rule, with constant offset for presynaptic spikes
**Spike pairing scheme**	All-to-all
**Pair-based update rule**	$\Delta w_+^{\mathrm{EI}} = \eta \exp(-\lvert\Delta t\rvert/\tau_{\mathrm{p}})$<br>$\Delta w_-^{\mathrm{EI}} = \eta \exp(\lvert\Delta t\rvert/\tau_{\mathrm{p}})$; $w^{\mathrm{EI}} = w^{\mathrm{EI}} - \eta \alpha_{\mathrm{ip}}$, for $t = t_i^*$
**Online update rule**	$\frac{dw_{ij}^{\mathrm{EI}}}{dt} = \eta \left(x_i(t) - \alpha_{\mathrm{ip}}\right) \delta\left(t - t_j^*\right) + \eta x_j(t) \delta\left(t - t_i^*\right)$

**F: Input**		
**Type**	**Target**	**Description**
**Poisson generator**	all E_i, I_i	Independent for each neuron, rate ν_{X}
**Patterned stimulus**	S_j for $j \in \{1 \cdots n\}$	Inhomogeneous Poisson process, rate $s_j(t)$

**G: Measurements**
Spiking activity

Supplementary Table 2. Simulation parameters.

A: Populations		
**Name**	**Value**	**Description**
N^{E}	8000	Excitatory population size
N^{I}	2000	Inhibitory population size
N_{u}	$\gamma_{\mathrm{u}} \times N^{\mathrm{E}}$	Stimulus-specific population size

B: Connectivity		
**Name**	**Value**	**Description**
ϵ	0.1	Connection probability (for EE, EI, IE and II connections)
γ	12	Scaling factor for the inhibitory synapses
$\bar{g}^{\mathrm{E}}$	1.8 nS	Excitatory synapse scaling factor
$\bar{g}^{\mathrm{I}}$	21.6 nS	Inhibitory synapse scaling factor ($\gamma\bar{g}^{\mathrm{E}}$)
g^{EE}	$w^{\mathrm{EE}}\bar{g}^{\mathrm{E}}$	Peak amplitude of excitatory conductance transient onto excitatory neurons (dynamic)
g^{EI}	$w^{\mathrm{EI}}\bar{g}^{\mathrm{I}}$	Peak amplitude of inhibitory conductance transient onto excitatory neurons (dynamic)
g^{IE}	$w^{\mathrm{IE}}\bar{g}^{\mathrm{E}}$	Peak amplitude of excitatory conductance transient onto inhibitory neurons (fixed)
g^{II}	$w^{\mathrm{II}}\bar{g}^{\mathrm{I}}$	Peak amplitude of inhibitory conductance transient onto inhibitory neurons (fixed)
$\bar{g}^{X}$	$\bar{g}^{\mathrm{E}}$	External input synapse scaling factor (and peak amplitude of conductance transient)
d	1.5 ms	Synaptic transmission delay

C: Neuron Model		
**Name**	**Value**	**Description**
τ_{m}	$\simeq$ 15 ms	Membrane time constant
C_{m}	250 pF	Membrane capacitance
Θ	-50 mV	Fixed firing threshold
V_{rest}	-70 mV	Resting membrane potential
V_{reset}	-60 mV	Reset potential
τ_{r}	5 ms	Absolute refractory period
g_{leak}	16.7 nS	Leak conductance
V_{E}	0 mV	Excitatory reversal potential
V_{I}	-80 mV	Inhibitory reversal potential

D: Synapse Model		
**Name**	**Value**	**Description**
τ_{E}	5 ms	Synaptic decay time constant for excitatory synapses
τ_{I}	10 ms	Synaptic decay time constant for inhibitory synapses

E: Plasticity Models		
**Name**	**Value**	**Description**
τ_{p}	20 ms	Coincidence time window (common for both STDP rules)
η	0.01	iSTDP learning rate
α_{ip}	0.12	Presynaptic offset
ρ_0	5spikes/s	Target firing rate
λ	0.01	eSTDP learning rate
α_{ep}	0.917556	Asymetry parameter

F: Input		
**Name**	**Value**	**Description**
ν_{X}	5 spikes/s	Rate of background stimulation
S	$\{\mathrm{A}, \mathrm{B}, \mathrm{C}\}$	Stimulus pattern set
σ_{u}	$[10, 50, 100]$	Peak rate of ‘burst’ spike pattern
N_{aff}	$[5, 10, 50, 100, 500]$	Number of afferent neurons
γ_{u}	$[0.01, 0.05, 0.1, 0.2, 0.3]$	Fraction of stimulus-specific input neurons

REFERENCES

Morrison, A., Diesmann, M., and Gerstner, W. (2008), Phenomenological models of synaptic plasticity based on spike timing, *Biological Cybernetics*, 98, 459–478, doi:10.1007/s00422-008-0233-1

Nordlie, E., Gewaltig, M.-O., and Plesser, H. E. (2009), Towards reproducible descriptions of neuronal network models., *PLoS computational biology*, 5, 8, e1000456, doi:10.1371/journal.pcbi.1000456

van Rossum, M. C., Bi, G.-Q., and Turrigiano, G. G. (2000), Stable Hebbian learning from spike timing-dependent plasticity., *The Journal of Neuroscience*, 20, 23, 8812–21

Vogels, T. P., Sprekeler, H., Zenke, F., Clopath, C., and Gerstner, W. (2011), Inhibitory plasticity balances excitation and inhibition in sensory pathways and memory networks., *Science*, 334, 6062, 1569–73, doi:10.1126/science.1211095
